# Development of a Novel Immune-Related Gene Signature to Predict Prognosis and Immunotherapeutic Efficiency in Gastric Cancer

**DOI:** 10.3389/fgene.2022.885553

**Published:** 2022-05-27

**Authors:** Dongliang Liu, Yuanmin Xu, Yu Fang, Kongwang Hu

**Affiliations:** ^1^ Department of General Surgery, The First Affiliated Hospital of Anhui Medical University, Hefei, China; ^2^ Department of General Surgery, The First Hospital Affiliated to the University of Science and Technology of China, Hefei, China

**Keywords:** gastric cancer, prognosis, immune-related gene signature, risk score, immunotherapy response

## Abstract

**Background:** Gastric cancer (GC) is the fifth most common malignancy and the third leading cause of tumor-related deaths globally. Herein, we attempted to build a novel immune-related gene (IRG) signature that could predict the prognosis and immunotherapeutic efficiency for GC patients.

**Methods:** The mRNA transcription data and corresponding clinical data of GC were downloaded from The Cancer Genome Atlas (TCGA) database as the training group and the GSE84437 data set as the testing cohort, followed by acquisition of IRGs from the InnateDB resource and ImmPort database. Using the univariate Cox regression analysis, an IRG signature was developed. Several immunogenomic analyses were performed to illustrate the associations between the immune risk score and tumor mutational burden, immune cell infiltrations, function of immune infiltration, clinical characteristics, immune subtype, and immunotherapeutic response.

**Results:** The analysis of 343 GC samples and 30 normal samples from the TCGA database gave rise to 8,713 differentially expressed genes (DEGs) and 513 differentially expressed immune-related genes (DEIRGs) were extracted. The novel IRG signature contained eight DEIRGs (FABP4, PI15, RNASE2, CGB5, INHBE, RLN2, DUSP1, and CD36) and was found to serve as an independent predictive and prognostic factor for GC. Then, the GC patients were separated into the high- and low-risk groups based on the median risk score, wherein the low-risk group presented a better prognosis and was more sensitive to immunotherapy than did the high-risk group. According to the time-dependent ROC curves and AUCs, the immunotherapeutic value of the signature was better than the Tumor Immune Dysfunction and Exclusion (TIDE) and T-cell inflammatory signature (TIS) scores. In addition, the AUCs of the risk score for predicting 1-, 2-, and 3-year OS were 0.675, 0.682, and 0.710, respectively, which indicated that the signature had great predictive power.

**Conclusion:** This study presents a novel IRG signature based on the tumor immune microenvironment, which could improve the prediction of the prognosis and immunotherapeutic efficiency for GC patients. The powerful signature may serve as novel biomarkers and provide therapeutic targets for precision oncology in clinical practice.

## Introduction

Gastric cancer (GC) is the fifth most common malignancy and the third leading cause of tumor-related deaths globally (Ferlay et al., 2019). Due to epigenetic changes, multiple genetic alterations, and the tumor microenvironment, GC is a complicated and highly heterogeneous disease, which results in variable prognosis in patients. Despite the significant improvement in treatment strategies and our understanding of cancer biology, a low 5-year survival rate of patients with advanced GC remains a challenge because of the late presentation, high metastasis, and recurrence rate (Siegel et al., 2021). Clinical outcomes in patients such as TNM stage, CA19-9, and CEA are primarily associated with risk factors that influence survival (Ning et al., 2018). However, there are still significant differences in the survival outcomes of patients with the same clinicopathological characteristics, which means that the current clinical outcome like the TNM staging system cannot reflect the intrinsic tumor heterogeneity. In addition, currently used evaluation measures based on transcriptome profiling, which are derived from different databases, and the diversity of the samples place restrictions on further clinical practice. Hence, further exploration of effective and stable models is an unmet medical need for predicting the prognosis of GC.

Recently, systemic immune therapeutics has become a practical option for patients with advanced GC, especially for patients without an opportunity to undergo radical resection (Kawazoe et al., 2021; Zeng et al., 2021). For instance, immunotherapy based on immune checkpoint blockade (ICB) has emerged as a new therapeutic treatment for several cancer types, which has certain promising application values in monotherapy or combination therapy (Anagnostou et al., 2017; Jiang et al., 2020). However, there are still plenty of patients who cannot benefit from immunotherapy due to immunotherapy resistance with the response rates to nivolumab and pembrolizumab (both PD-1/PD-L1 inhibitors) being less than 50% (Ando et al., 2021). The tumor microenvironment, defined as the tissue environment surrounding the tumor, consists of stromal cells, cytokines and chemokines, extracellular matrix, immune cells, and other secretory molecules. Increasing evidence has demonstrated that infiltrating immune cells in the tumor microenvironment play a pivotal role during cancer initiation, aggressiveness, and therapeutic response (Gentles et al., 2015; Dong et al., 2021; Paijens et al., 2021). And immune infiltration was utilized by cancer cells to escape the immune surveillance and elimination. Dai et al. (2016) conducted a comprehensive immunohistochemistry analysis of the tumor microenvironment and investigated that induction of immune checkpoints within GC patients reflects a high immune infiltration density. A further study found that the high level of infiltrated macrophages released proinflammatory cytokines such as IL-6 and TNF-ɑ, which induced PD-L1 expression in cancer cells (Ju et al., 2020). Previous studies have identified several survival-related biomarkers in GC cancer for risk stratification, but the depth and existing work of these studies are not satisfactory.

Recently, several studies have built numerous gene signatures to predict survival stratification in GC (Jiang et al., 2019; Liu et al., 2020a; Qiu et al., 2020; Yu et al., 2020). Unfortunately, due to the lack of statistical comparison with other models like the Tumor Immune Dysfunction and Exclusion (TIDE) and T-cell inflammatory signature (TIS) systems, this might reduce the effectiveness and robustness of the gene model. In the present study, we identify eight differentially expressed and survival-associated immune-related genes (IRGs) to construct and validate a novel immune-related prognostic signature for GC patients. In training and testing cohorts of our study, this signature seems to serve as an independent predictive and prognostic factor. Moreover, the results of our study suggest that the immunotherapeutic value of our signature is better than those of the TIDE and TIS scores. Furthermore, we explored the potential predictive role of the signature in immunotherapy efficacy. This comprehensive immune-based signature might deepen our understanding of the association between prognosis and immune infiltration and the function of immune infiltration and tumor mutation burden (TMB) in GC, and may be useful in laying the foundation for the clinical management and targeted therapy of GC.

## Materials and Methods

### Data Selection

RNA sequencing data, probe annotation files, and the corresponding clinical data of GC were downloaded from The Cancer Genome Atlas (TCGA, https://portal.gdc.cancer.gov/) database as the training group, which also included the normal samples. The mRNA transcription data and matching clinical data of GC were extracted from the Gene Expression Omnibus (GEO, https://www.ncbi.nlm.nih.gov/geo/) database as a testing cohort. All samples included the overall survival rate and survival status data. The Immunology Database and Analysis Portal (ImmPort) and InnateDB resource provided human immunological data and the immune-related genes (IRGs) lists. Since the patients’ data used in the present study were retrieved from public databases, informed consent was not required.

### Differential Expression Gene Extraction and Functional Enrichment Analysis

The “limma” package was used to extract DEGs from TCGA with criteria as follows: log_2_FC >1 and adjusted *p* < 0.05. Moreover, the DEIRGs were developed from the intersection of the DEGs and IRGs lists. Visualization of DEGs and DEIRGs were drawn by volcano plots and heat maps using the “pheatmap” and “ggplot2” packages, respectively. Functional enrichment analysis of the identified genes based on the Gene Ontology (GO) and Kyoto Encyclopedia of Genes and Genomes (KEGG) pathways was conducted by using the “ggplot2,” “cluster Profiler,” and “enrich plot” packages. The GO terms predicted the functional effect of the target genes on account of three aspects, namely, molecular functions (MF), biological processes (BP), and cellular components (CC).

### Identification of Co-Expression Modules and Gene–Gene Interaction Network Analysis

The weighted gene co-expression network analysis (WGCNA) package was performed to identify co-expression genes and modules based on the DEIRGs expression profiles. The soft thresholding power was chosen based on the scale-free topology criterion, and Pearson correlation coefficients were calculated for all gene comparisons. The adjacency matrix was calculated into a topological overlap matrix to construct the highly interconnected gene cluster dendrogram. Finally, the genes with similar expression profiles were sorted into the same module. Then, the module genes those were most associated with the sample status were included for the gene–gene interaction network and further analysis.

### Establishment and Validation of the Immune-Related Risk Signature

The univariate Cox regression analysis was carried out to identify the survival-related DEIRGs (*p* < 0.05), and the result was visualized as a forest plot. Besides, the genetic alteration details of survival-related DEIRGs were also assessed by using the “maftools” package. Multivariate Cox regression was performed to establish the IRG prognostic signature using the training group. The established signature is as follows: risk score = gene A expression × coefficient A + gene B expression × coefficient B + … + gene N expression × coefficient N, and the testing cohort was used to validate the result. Then, the GC patients were separated into the high- and low-risk groups based on the median risk score. We used Kaplan–Meier survival analysis to confirm the prognostic value of the established signature. In addition, univariate and multivariate Cox regression analyses were conducted to investigate if the risk score is an independent predictive and prognostic factor.

### Gene Set Enrichment Analysis

To investigate the underlying molecular mechanism of immune-related signature, the gene set enrichment analysis (GSEA) was used to identify the KEGG pathways predicted to be associated with the risk score with an FDR <0.05. Subsequently, the top five KEGG pathways those were most related to the risk score were selected in the high- and low-risk groups, respectively.

### Mutation Analysis

The mutation data containing somatic variants were retrieved from the TCGA. The TMB counts for each GC sample were measured. Differences in TMB were compared between the high- and low-risk groups, and we used OncoPrint and boxplot to display the findings. Besides, we explored the correlation between TMB and the signature risk score using the “limma,” “ggplot2,” “ggpubr,” and “ggExtra” packages.

### Immune Cell Infiltration and Function of Immune Infiltration Analysis

The infiltration of immune subpopulations of the high- and low-risk groups was estimated using CIBERSORT, and the immune cells with *p* < 0.05 were identified in the two groups. In addition, to analyze the link between the function of immune infiltration and risk score in GC, we recognized the differential function of immune infiltration among immune functional data sets. Subsequently, the Kaplan–Meier curve analyses were adopted to assess the OS of immune cell infiltration and the function of immune infiltration between the high- and low-infiltration groups.

### Analyses of Correlation Between Risk Score and Clinical Characteristics

To analyze the correlation between the risk score and clinical characteristics such as age, gender, grade, TNM status, T stage, N stage, and M stage in GC, we used the “ComplexHeatmap” package and strip chart to show the relationships. We also explored the association between the risk score and immune subtype.

### Clinical Utility of the Immune-Related Risk Signature

To verify the therapeutic benefits of the risk score calculated by our model, the violin plot illustrated the risk score distributions for patients with GC in the Tumor Immune Dysfunction and Exclusion (TIDE), T-cell dysfunction, and T-cell exclusion scores. Time-dependent ROC curves of immunotherapy response prediction at 1-, 2-, and 3-year survival rates based on the risk score for GC were calculated. The ROC curves of our signature compared with the TIDE and T-cell inflammatory signature (TIS) scores were used to validate the clinical utility of the immune-related risk signature and visualized as AUC values.

### Validation of the Core Differentially Expressed Immune-Related Genes

Kaplan–Meier log-rank tests were implemented to validate the prediction ability and prognostic value of the core DEIRGs in our samples. Besides, to further verify the differential expression and prognostic value of the core DEIRGs, we used an online database called TIMER and Kaplan–Meier plotter, which contained survival information and expression data of GC patients. Immunohistochemical (IHC) staining evaluation of the eight survival-related DEIRGs protein expression in clinical samples of GC patients with normal and tumor tissues was performed using the Human Protein Atlas.

## Results

### Identification of DEGs and Differentially Expressed Immune-Related Genes in Gastric Cancer

**FIGURE 1:**
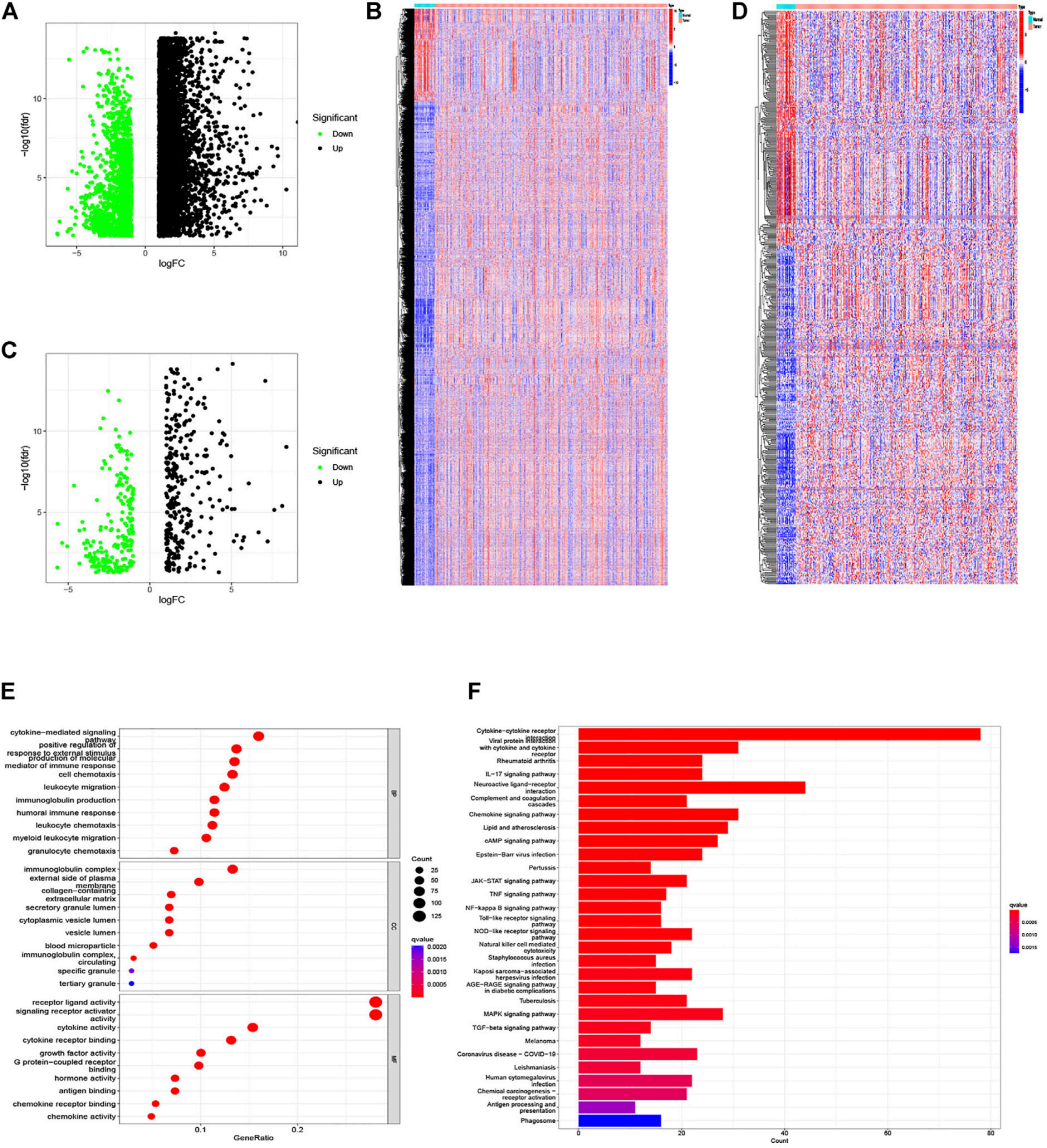
DEGs and differentially expressed IRGs between GC and normal tissues, and functional enrichment analysis of differentially expressed IRGs. **(A,B)** The volcano plot and heat map of DEGs in GC samples from the TCGA database. **(C,D)** The volcano plot and heat map of IRGs differentially expressed in our samples. **(E)** Barplot of GO enrichment. **(F)** Barplot of KEGG enriched terms.

The whole analysis process was presented in Supplementary Figure S1 to identify an IRG signature. The analysis of 343 GC samples and 30 normal samples from the TCGA database gave rise to 8,713 DEGs using the R software “limma” package (Figure 1A,B), and 513 DEIRGs were extracted from the intersection of the DEGs and IRGs lists (Figure 1C,D). Subsequently, functional enrichment analysis of the identified genes based on the GO and KEGG pathway were conducted. The most enriched terms in the GO analysis for BP, CC, and MF were cytokine-mediated signaling pathway, immunoglobulin complex, and receptor–ligand activity (Figure 1E). Moreover, the KEGG pathways analysis illustrated that the cytokine–cytokine receptor interaction, viral protein interaction with cytokine and cytokine receptor, and rheumatoid arthritis were the top three enriched in DEIRGs (Figure 1F).

### Identification of Co-expression Modules

**FIGURE 2:**
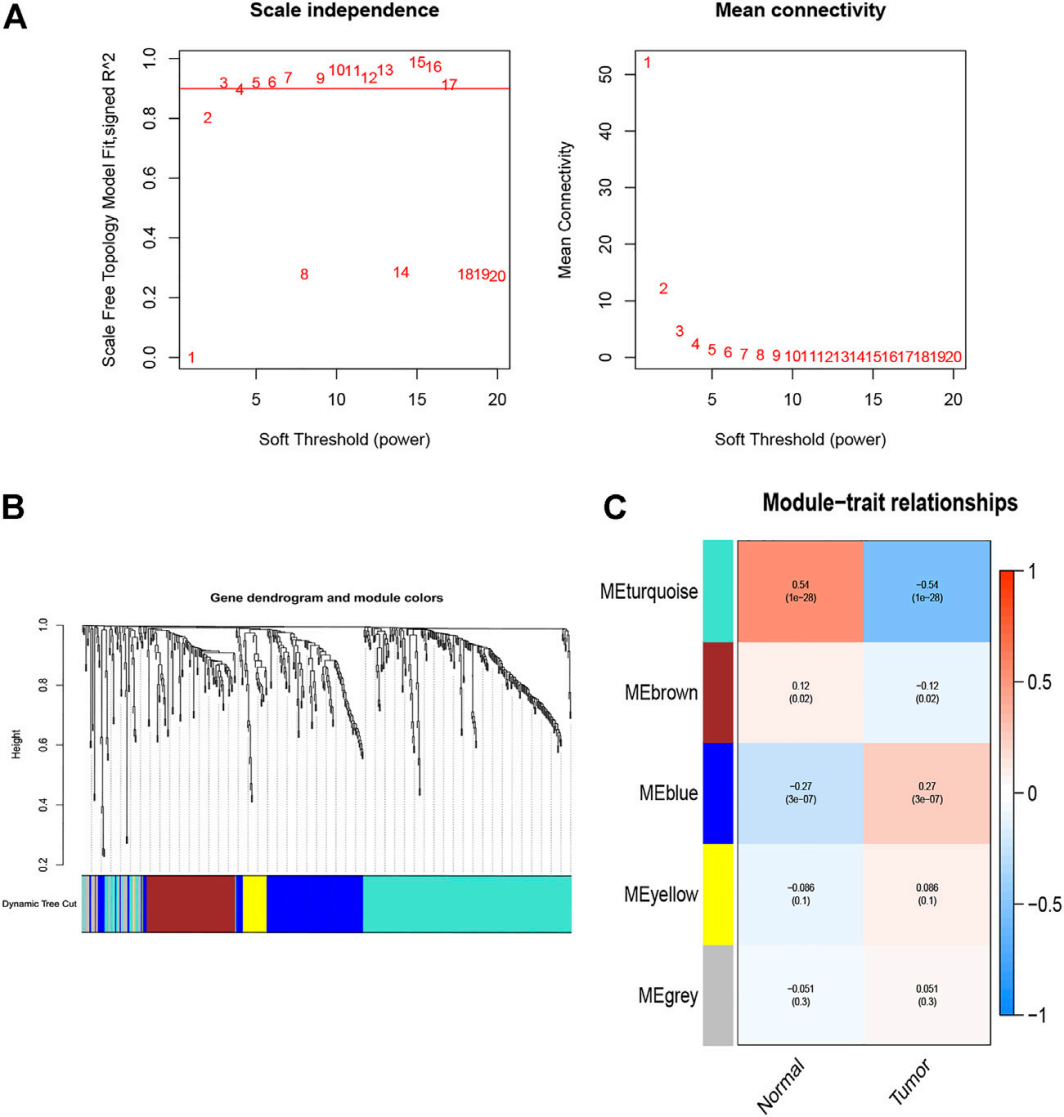
Identification of co-expression modules. **(A)** Network topology analysis for various soft-thresholding powers, the suitable soft-thresholding power of β = 5 was identified. **(B)** The gene dendrogram and the corresponding module colors. Samples were clustered by the dynamic cut tree algorithm, and height indicates the Euclidean distance. **(C)** The module–trait relationships between tumor and normal tissues.

**FIGURE 3:**
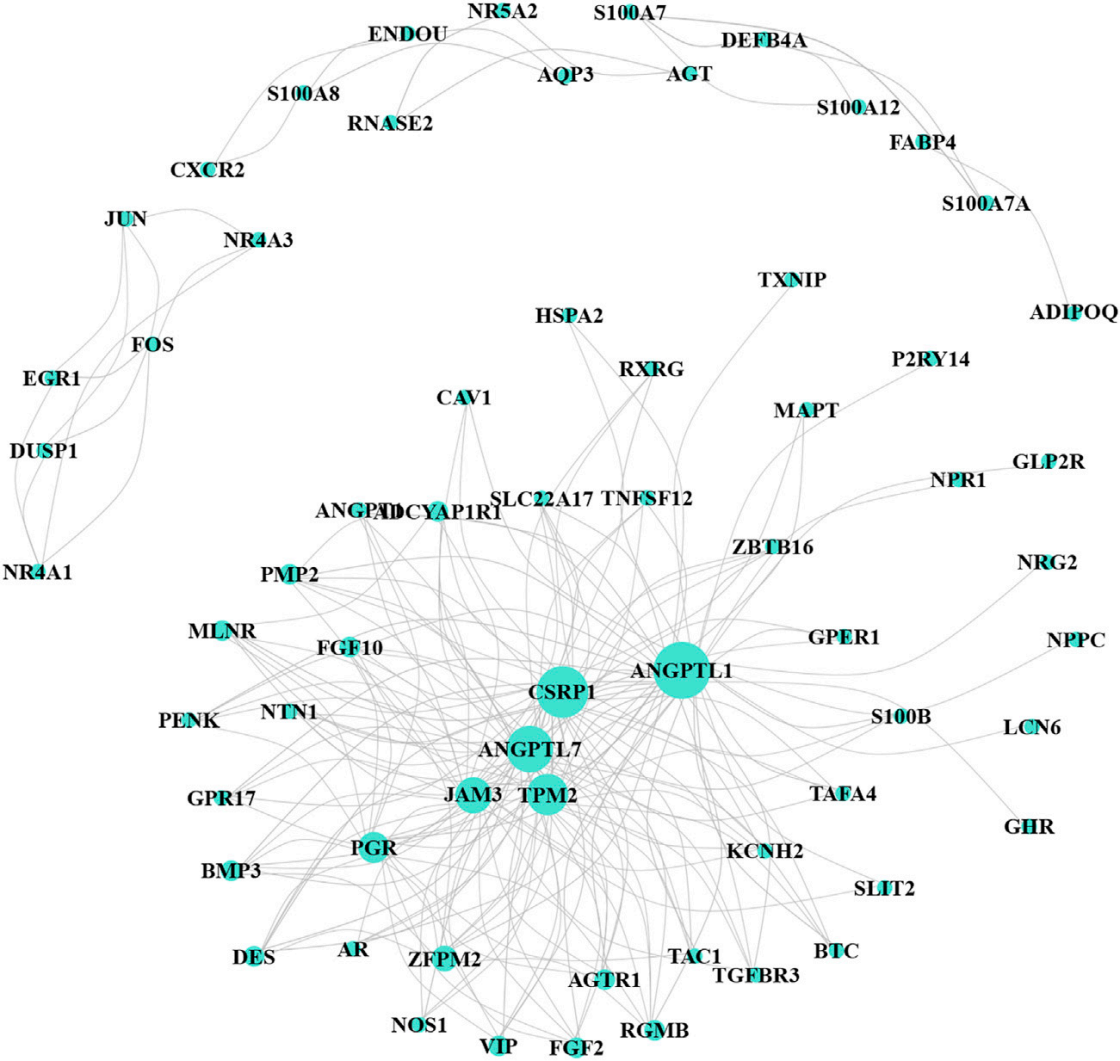
Co-expression network for turquoise-module genes. The size of the node represents the number of interaction proteins; lines indicate correlations.

The WGCNA R package was performed to identify co-expression genes and modules based on the DEIRGs expression profiles. Here, the suitable soft-thresholding power of *β* = 5 was identified ensuring close to the scale-free network (Figure 2A). After the samples were clustered by the dynamic cut tree algorithm, the eigenvectors of each module were measured (Figure 2B). Then, we merged these closed modules into new modules, and a total of five modules (turquoise, brown, blue, yellow, and gray modules) were screened for the DEIRGs in the end (Figure 2C). Finally, the turquoise-module containing 240 DEIRGs most associated with the sample status was utilized for the gene–gene interaction network and further analysis (Figure 3).

### Establishment and Validation of the Eight-Gene–Based Immune-Related Signature

**FIGURE 4:**
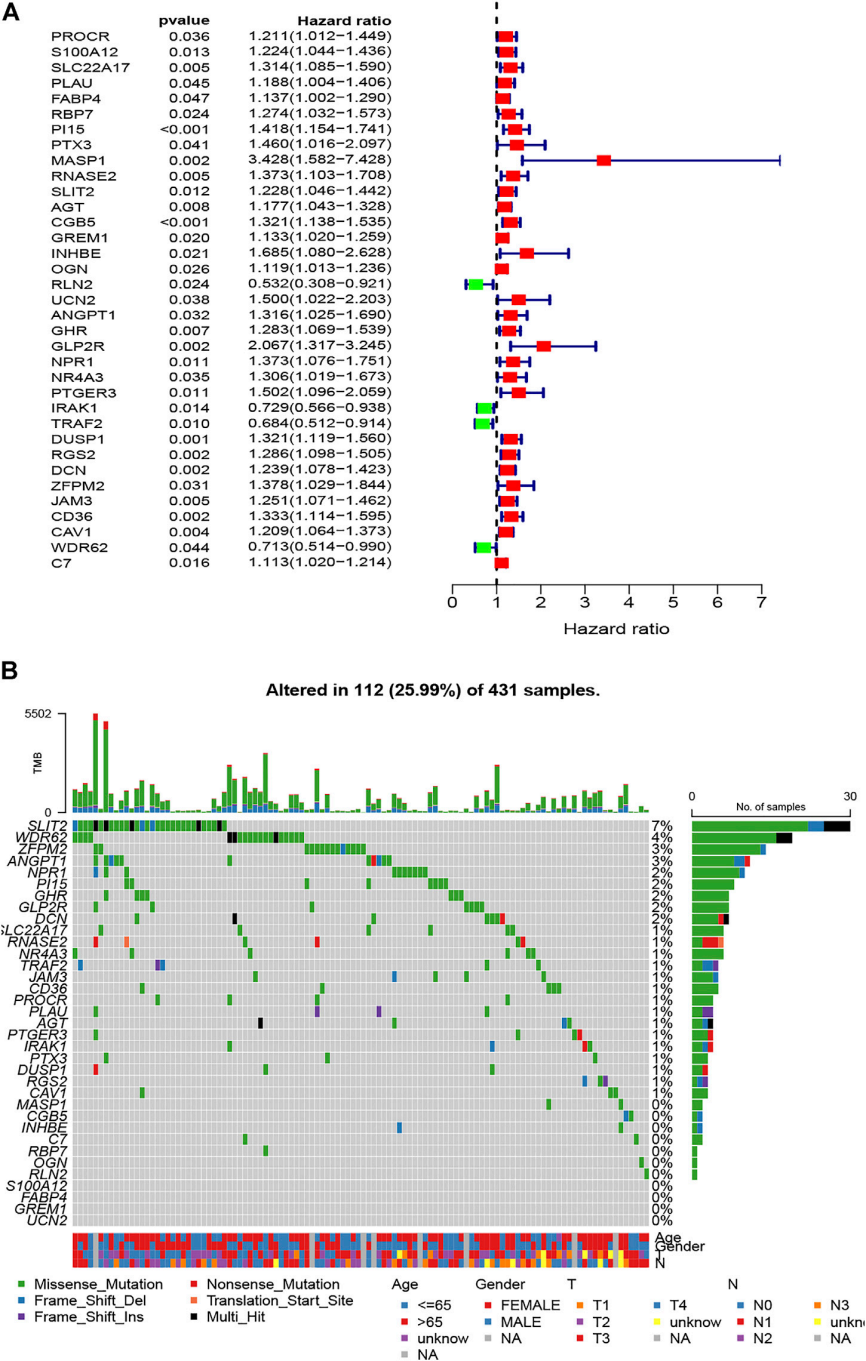
Identification of survival-related DEIRGs by univariate Cox regression analysis. **(A)** The forest plot of survival-related DEIRGs. **(B)** Genetic alterations of survival-associated DEIRGs.

**FIGURE 5:**
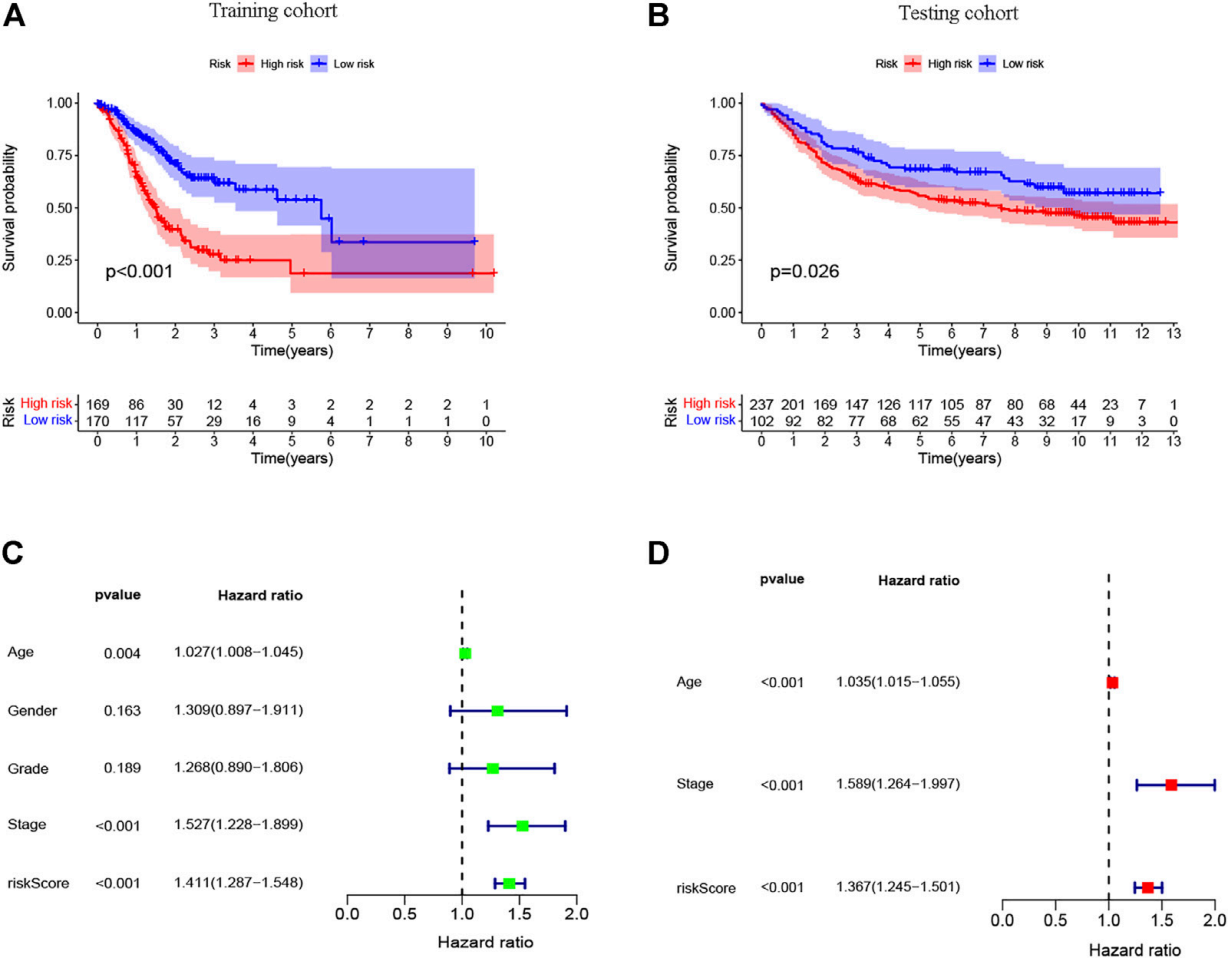
Survival analysis of the prognostic immune signature in the **(A)** training (TCGA) and **(B)** testing (GEO) cohorts, and independent prognostic analysis of the immune signature. **(C)** The univariate and **(D)** multivariate Cox regression analysis of clinical parameters and risk score.

After further filtering, the 35 survival-related DEIRGs were verified by conducting the univariate Cox regression analysis based on the 240 DEIRGs (Figure 4A). Besides, the genetic alteration details of the survival-associated DEIRGs are shown in Figure 4B. The multivariate Cox regression analysis was then performed to establish the prognostic signature. An eight-gene–based immune-related signature was acquired as follows: risk score = −mRNA expression level of FABP4 × 0.228 + mRNA expression level of PI15 × 0.201 + mRNA expression level of RNASE2 × 0.219 + mRNA expression level of CGB5 × 0.318 + mRNA expression level of INHBE × 0.720 − mRNA expression level of RLN2 × 0.674 + mRNA expression level of DUSP1 ×0.206 + mRNA expression level of CD36 × 0.315. The GC patients were separated into the high- and low-risk groups based on the median risk score. The Kaplan–Meier survival analysis clarified that the OS was significantly longer in the low-risk group in the training and testing cohorts (*p* < 0.05; Figures 5A,B).

### The Eight-Gene–Based Signature Can Serve As an Independent Predictive and Prognostic Factor

To explore the independence of the eight-gene–based signature, univariate and multivariate Cox analyses were performed using the entire TCGA cohort. The univariate analysis demonstrated that age, stage, and the risk score were independently associated with the OS in GC (*p* < 0.05) (Figure 5C), and the results showed that the risk score could be an independent survival predictor of the OS in the multivariate analyses (*p* < 0.001) (Figure 5D). Thus, this signature may serve as an independent predictive and prognostic factor.

### Gene Set Enrichment Analysis and Tumor Mutation Burden Analysis Correlated With the Risk Score

**FIGURE 6:**
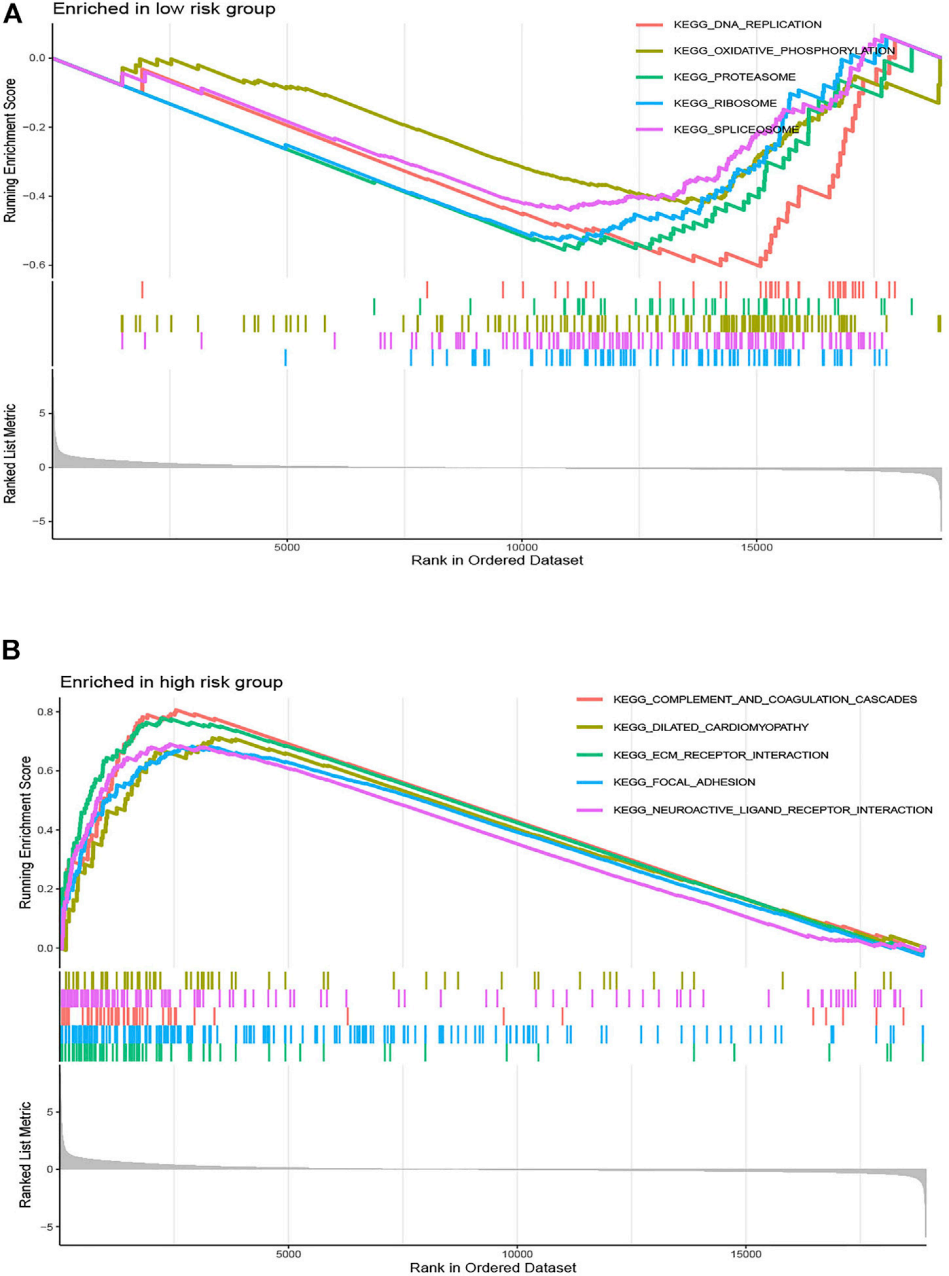
Gene set enrichment analysis correlated with the risk score. Gene set enrichment analysis of the top five pathways significantly enriched in **(A)** the low-risk group and **(B)** the high-risk group.

**FIGURE 7:**
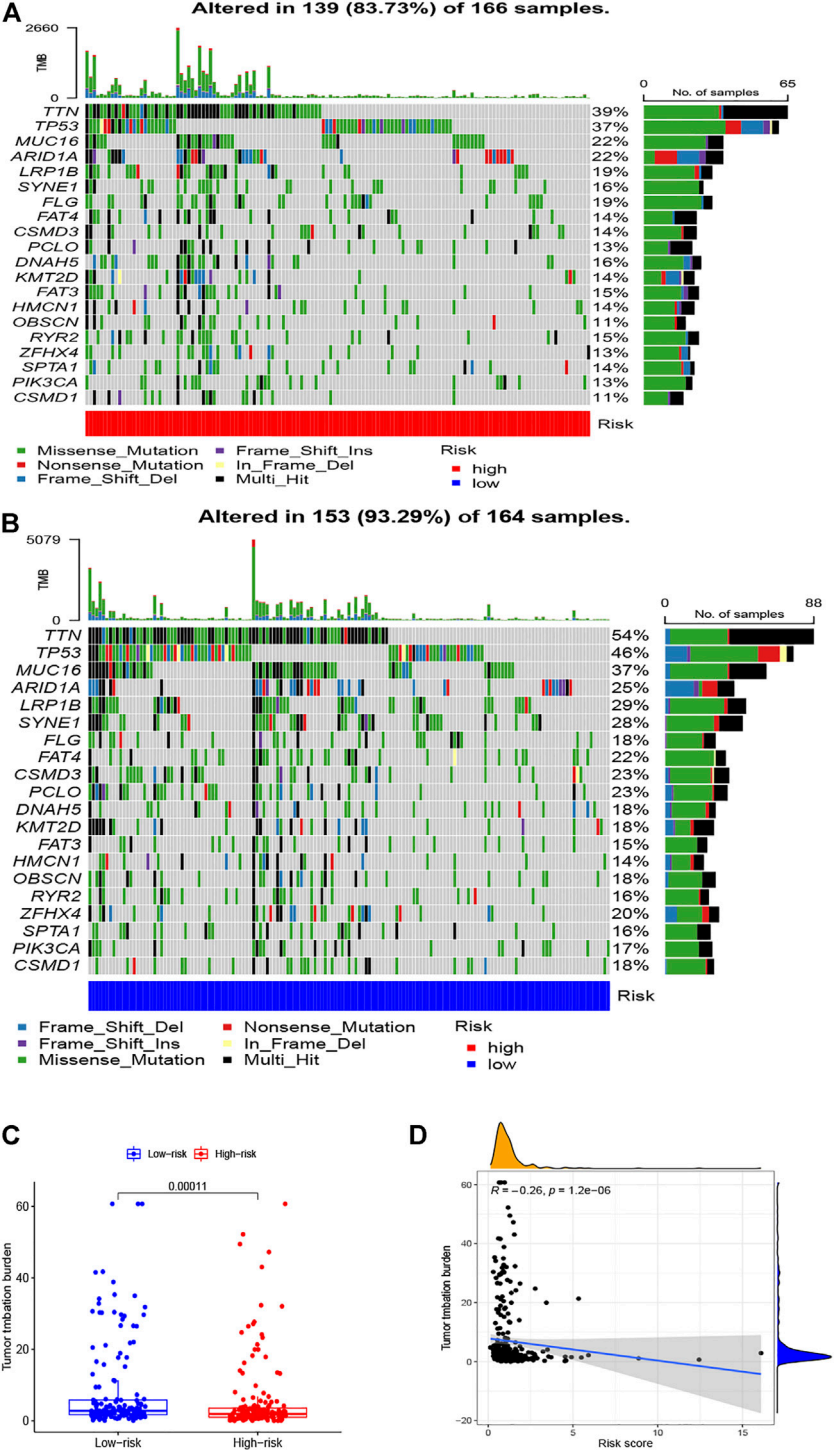
Correlations between TMB and risk scores. OncoPrint displays the mutation profile of the top 20 frequently mutated genes in **(A)** high- and **(B)** low-risk patients. **(C)** Boxplot shows the difference in TMB between high- and low-risk patients. **(D)** The correlation analysis between TMB and risk scores.

To illustrate the underlying molecular mechanism of immune-related signature, we performed the GSEA to identify the KEGG pathways with the FDR <0.05. The top five KEGG pathways most related to the risk score were selected, namely, DNA replication, oxidative phosphorylation, proteasome, ribosome, and spliceosome in the low-risk group and complement and coagulation cascades, dilated cardiomyopathy, ECM receptor interaction, focal adhesion, and neuroactive ligand–receptor interaction in the high-risk group (Figure 6). The landscape of the TMB profile in GC from the whole TCGA database is shown in Supplementary Table S1, and the mutation profile of the top 20 frequently mutated genes was identified in high- (Figure 7A) and low-risk (Figure 7B) patients. We found that TMB was significantly higher in the low-risk group than in the high-risk group (*p* = 0.00011, Figure 7C). In addition, we consequently measured the correlation between the risk score and TMB and found that TMB had a significant negative correlation with the risk score (R = −0.26, *p* = 1.2e - 06, Figure 7D).

### Estimation of Immune Cell Infiltration and Function of Immune Infiltration Based on the Risk Score

**FIGURE 8:**
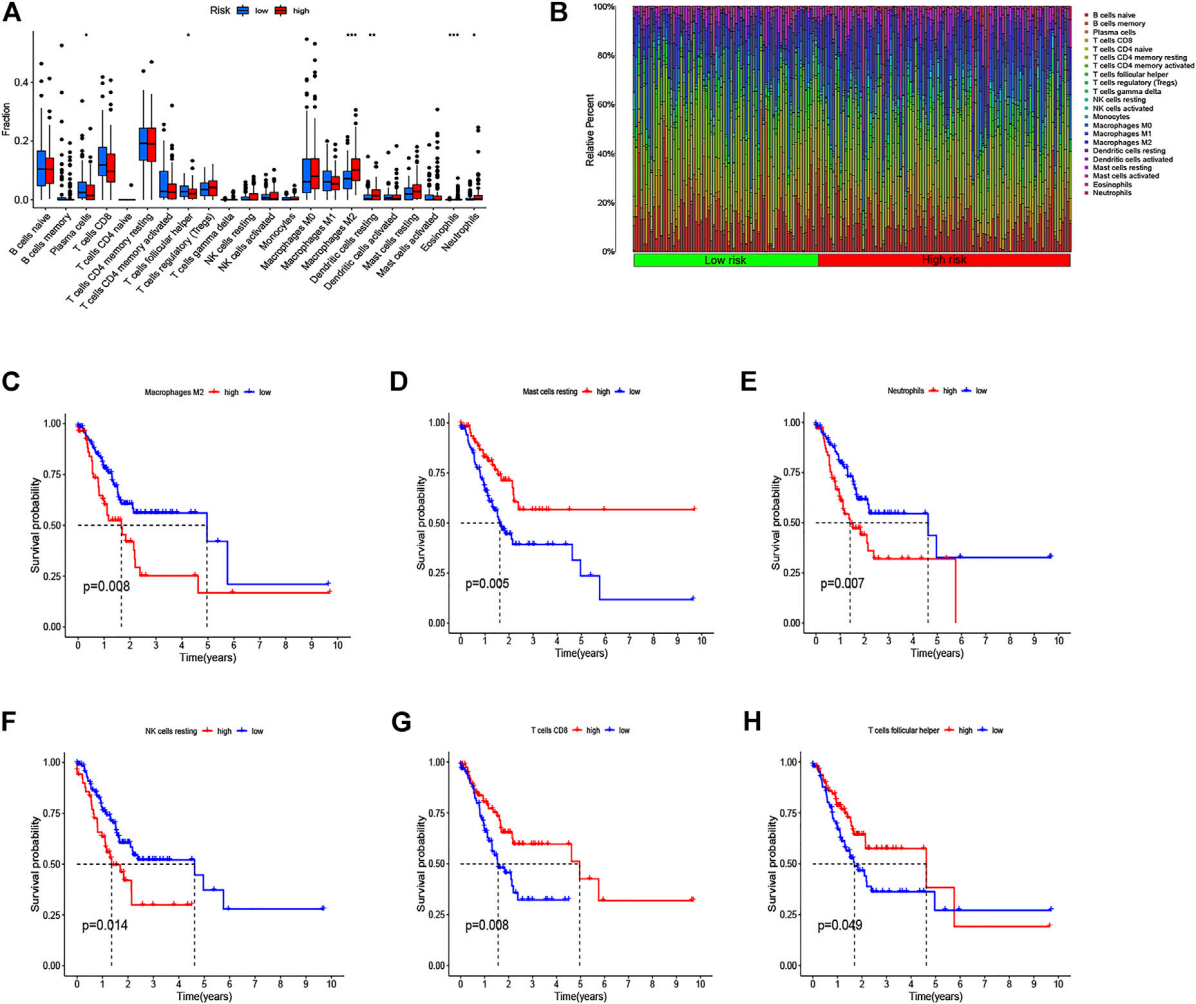
Analysis of immune cell infiltration and the Kaplan–Meier curve for survival based on immune cell. **(A)** Infiltration level of immune cells in the high-risk and low-risk groups. **(B)** Barplot shows the distribution of 22 immune cells in the high-risk and low-risk groups. **(C–H)** Kaplan–Meier survival curve shows the relationship between the six immune cells and survival with *p* < 0.05.

**FIGURE 9:**
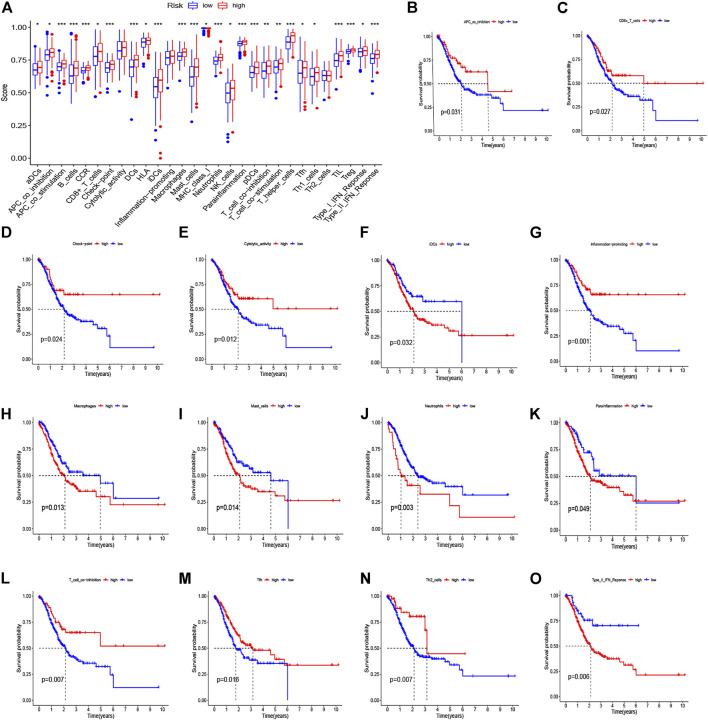
Immune function–related analyses between high- and low-risk groups. **(A)** The boxplot for 29 immune functions in high- and low-risk groups. **(B–O)** Kaplan–Meier curve for survival in high- and low-infiltration groups based on 14 significant immune functions.

We used CIBERSORT to identify the proportion of 22 immune cells of the high- and low-risk groups to investigate the immune infiltration in each TCGA sample, and the six immune cells with *p* < 0.05 were selected (Figure 8A). We found that the infiltration level of macrophages M2 cells, dendritic cells resting, plasma cells, follicular helper T cells, eosinophils, and neutrophils was significantly correlated with the risk score. Specifically, GC patients with high-risk scores had a higher proportion of macrophages M2 cells than did the low-risk group. Also, the barplot showed the distribution of 22 immune cells in the high- and low-risk groups (Figure 8B). In addition, the Kaplan–Meier curve analyses were adopted to assess the OS of immune cells between the high- and low-infiltration groups, and the results indicated that six immune cells were significantly related to the prognosis (Figures 8C–H). Meanwhile, we also explored the relationship between the risk score and function of immune infiltration of GC patients from the TCGA-CESC cohort. As depicted in Figure 9A, the infiltration of 29 immune functions of the high- and low-risk groups was estimated, and 26 immune functions were found significantly associated with the risk score. The Kaplan–Meier plots demonstrated that 14 functions of immune infiltration had prognostic values in the high- and low-infiltration groups. In addition, the results from the TIMER database showed that the expression level of the eight genes had a good correlation with the infiltrating immune cells in the tumor microenvironment (Supplementary Figure S2).

### Relationship Between Clinical Characteristics, Immune Subtype, and the Risk Score

**FIGURE 10:**
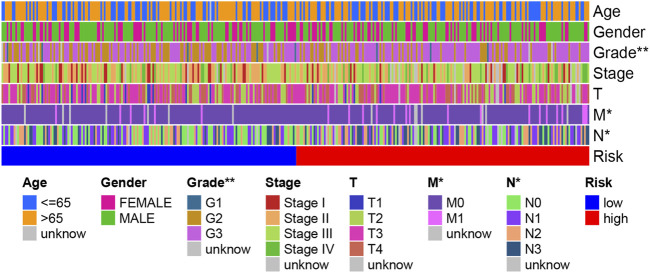
A strip chart showing the grade; N stage and M stage were significantly associated with the risk score.

We performed a relationship analysis comparing the risk score and clinical characteristics, which indicated that the risk score was associated with grade (*p* < 0.01), N stage (*p* < 0.05), and M stage (*p* < 0.05) (Figure 10). Furthermore, we also verified the relationship between the risk score and immune subtype. However, there was no significant association between the risk score and immune subtype (*p* > 0.05) (Supplementary Figure S3).

### Comparison of the Eight-Gene–Based Signature and Other Models

**FIGURE 11:**
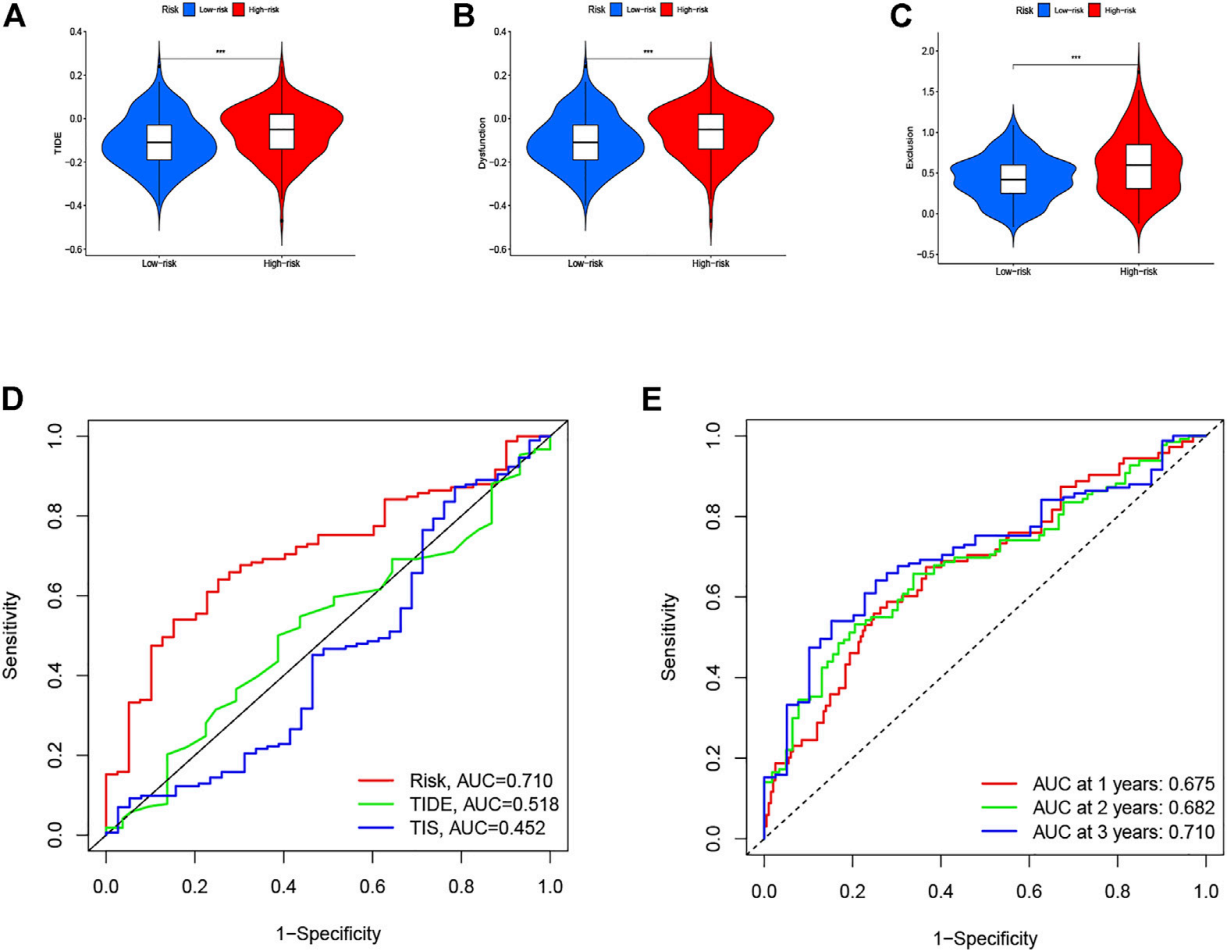
Therapeutic benefits of risk scores calculated by our model. Violin plot illustrates the risk score distributions for patients with GC in **(A)** TIDE score, **(B)** T-cell dysfunction score, and **(C)** T-cell exclusion score. **(D)** ROC curves of our model compared with TIDE and TIS scores. **(E)** Time-dependent ROC curves of immunotherapy response prediction at 1-, 2-, and 3- year survival rates based on the risk scores for GC.

Herein, the TIDE, T-cell dysfunction, and T-cell exclusion scores were generated from the TIDE system. To access the value of the eight-gene signature in tumor immunotherapy, we analyzed the correlation between the risk score and the TIDE, T-cell dysfunction, and T-cell exclusion scores. In the present study, as shown in Figures 11A–C, the patients in the low-risk group displayed lower TIDE, T-cell dysfunction, and T-cell exclusion scores than did patients in the high-risk group (*p* < 0.001). To further validate the identified signature, we utilized the TIS score in the high- and low-risk groups. Time-dependent ROC curves and AUCs were plotted to determine the clinical utility of the signature when compared with the TIDE and TIS scores. The AUC value for the identified signature in the TCGA data set was 0.710 (Figure 11D). The result suggested that the immunotherapeutic value of our signature was better than that of the TIDE and TIS scores. In addition, the AUCs of the risk score for predicting 1-, 2-, and 3-year OS were 0.675, 0.682, and 0.710, respectively (Figure 11E). The abovementioned findings indicate that this signature has good predictive power.

### Validation the Core Differentially Expressed Immune-Related Genes

**FIGURE 12:**
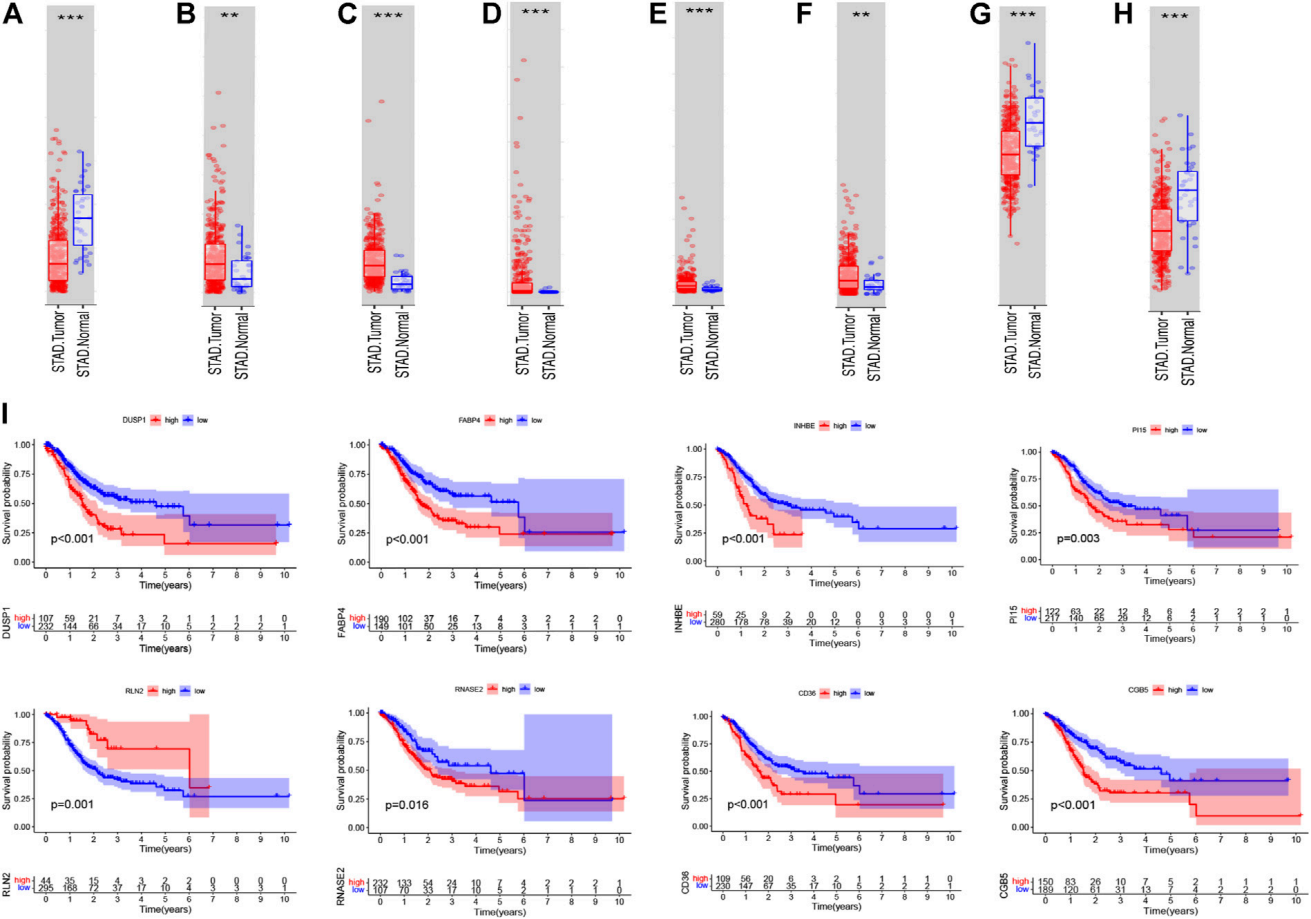
Differential expression; Kaplan–Meier survival curve of all GC patients with the eight immune-related genes. **(A–H)** Boxplots showing the relative expression levels of FABP4 **(A)**, PI15 **(B)**, RNASE2 **(C)**, CGB5 **(D)**, INHBE **(E)**, RLN2 **(F)**, DUSP1 **(G)**, and CD36 **(H)** in normal tissues and GC tissues. ***p < 0.001 and **p < 0.01. **(I)** Survival curves of GC patients with high expression and low expression of the following genes: FABP4, PI15, RNASE2, CGB5, INHBE, RLN2, DUSP1, and CD36.

After we had illustrated the immunotherapeutic value of the eight-gene–based immune-related signature, we next explored the differential expression and prognostic value of the core DEIRGs. The results reveal that the mRNA levels of PI15, RNASE2, CGB5, INHBE, and RLN2 were significantly higher in GC than in normal tissues. Besides, FABP4, DUSP1, and CD36 were confirmed to have a lower expression in GC than in normal tissues (Figures 12A,H). Immunohistochemistry (IHC) was used to examine the protein expression of FABP4, PI15, RNASE2, CGB5, INHBE, and CD36 in normal and GC tissues (Supplementary Figure S4). The association between the eight-DEIRG profile and OS of GC patients is displayed in Figure 12I. The results revealed that GC patients with a higher expression of FABP4, PI15, RNASE2, CGB5, INHBE, DUSP1, and CD36 showed significantly poorer OS than those with low expression, while patients with a higher expression of RLN2 showed the opposite trend. Not surprisingly, the prognostic value of the core DEIRGs mRNA expression was further verified by Kaplan–Meier plotter (Supplementary Figure S5).

## Discussion

Although the past 20 years had been characterized by the expansion of clarifying the molecular mechanism of GC and an advance in diagnostic and therapeutic methods for managing GC patients, the survival outcomes have remained poor (Liu et al., 2020b; Sugawara et al., 2021). Immunotherapy has emerged as a new therapeutic treatment for several cancer types, and the utilization of gene expression signatures to determine the immunotherapeutic outcomes is yet to be reported. Guo and Jing (2021) identified a five-gene risk model with excellent stability and reliability for predicting prognosis in breast cancer patients. Similarly, they had discovered an immune-related lncRNA signature related to the prognosis and immunotherapeutic efficiency of bladder cancer patients (Zhang et al., 2020). These findings suggest that pivotal DEIRGs' correlation with the tumor microenvironment may serve as prognostic signatures and could help devise individualized immunotherapies. In the present study, we developed an immune-related signature composed of only eight genes, and to the best of our knowledge, our signature is the first panel described to date, which allows precise estimation of both the survival outcomes and immunotherapeutic efficiency in GC patients.

In the present study, we identified a robust risk score signature comprising eight genes, namely, FABP4, PI15, RNASE2, CGB5, INHBE, RLN2, DUSP1, and CD36. Previous studies have described certain associations between the genes and tumorigenesis and pathogenesis of cancer. For instance, Gyamfi et al. (2021) indicated that increased CD36 expression activates the STAT3 signal pathway required for adipocyte-induced epithelial–mesenchymal transition and stemness in adipocyte–breast cancer, and together with FABP4 regulates fatty acid import. Gao et al. (2016) demonstrated that CGB5 may induce tumor growth and vasculogenic mimicry formation *via* activation of the luteinizing hormone receptor signal pathway. Ren et al. (2015) found that RLN2 facilitates cell proliferation, migration, and invasiveness of osteosarcoma *in vitro* through the S100A4/MMP-9 signal pathway. In addition, to verify the prognostic value of these genes, Kaplan–Meier log-rank tests were implemented and the results revealed that GC patients with higher expression of FABP4, PI15, RNASE2, CGB5, INHBE, DUSP1, and CD36 showed significantly poorer OS than those with low expression, while patients with a higher expression of RLN2 showed the opposite trend. Not surprisingly, the prognostic value of these genes was further validated through the Kaplan–Meier plotter. Based on the relatedness of the tumor, these eight genes might serve as predictive and prognostic biomarkers in clinical practice and precise therapeutic targets for GC patients.

GC is a complicated and highly heterogeneous disease due to epigenetic changes, multiple genetic alterations, and the tumor microenvironment, and the frequency of genetic alterations varies significantly in tumor cells (Chalmers et al., 2017). TMB, which mirrors the somatic mutation frequency, has been reported to be correlated with response to immune checkpoint inhibitors (ICIs) (Wu et al., 2020). We analyzed the genetic alteration in GC patients and found that the most frequently mutated genes included TTN, TP53, and MUC16 in both the high- and low-risk groups. In addition, we found that TMB was significantly higher in the low-risk group than in the high-risk group, and patients in the low-risk group displayed lower TIDE, T-cell dysfunction, and T-cell exclusion scores. The TIDE score is a computational method to evaluate two primary mechanisms of tumor immune escape, namely, the induction of T-cell dysfunction in tumors with high infiltration of cytotoxic T lymphocytes and the prevention of T-cell infiltration in tumors with low cytotoxic T-lymphocytes level (Jiang et al., 2018; Fu et al., 2020). A lower TIDE score reflects a lower potential for immune evasion, which indicates that the patients had a more favorable response to the ICB and are more likely to benefit from anti-PD-1 and anti-CTLA-4 immunotherapy. Beforehand, the Kaplan–Meier survival analysis clarified that the OS was significantly longer in the low-risk group in the training and testing cohorts. Consistent with our results, Stenzinger et al. (2019) demonstrated that the high TMB status was associated with increased patient response to ICIs and prognosis benefits from immunotherapy. A recent study on hepatocellular carcinoma also reported that the more sensitive to ICIs group had a significantly higher TMB than the group with less sensitive to ICIs (Xu et al., 2019). Besides, we consequently measured the correlation between the risk score and TMB and found that TMB had a significant negative correlation with the risk score. Thus, TMB may serve as a prognostic prediction in ICIs immunotherapy, and the detection of the somatic mutation frequency before initiating immunotherapeutic treatment is necessary.

Recently, many studies focused on the immune landscape have brought more attention to the molecular mechanism in cancer research. Immune cells infiltration is a significant characteristic of the tumor microenvironment, which has been shown to play an important role in the progression of tumors. Therefore, we used CIBERSORT to identify the proportion of 22 immune cells in the low- and high-risk groups to investigate immune infiltration in each sample and found that the infiltration level of macrophages M2 cells, dendritic cells resting, plasma cells, T cells follicular helper, eosinophils, and neutrophils were significantly correlated with the risk score. In addition, Kaplan–Meier curve analyses were adopted to assess the OS of immune cells between the high- and low-infiltration groups, and the results have indicated that macrophages M2 cells, T cells follicular helper, and neutrophils were significantly related to prognosis. We first illustrated the correlation between immune cells infiltration and prognosis distribution of immune cells in GC patients separated by the identified signature. First, high densities of M2 macrophages were found in the high-risk group, which were correlated with poor prognosis. Macrophages contain different subtypes, including M1 and M2 macrophage phenotypes, which are generally considered to directly or indirectly promote tumor proliferation and metastasis in GC and are positively correlated with the invasion depth and tumor stage (Ishigami et al., 2003). The M1 macrophages can stimulate apoptosis and suppress proliferation and the development of neovascularization, while M2 macrophages can accelerate both cancer growth and metastasis (Wu et al., 2015; Wang et al., 2016). In recent years, researchers have expanded their studies to figure out both M1 and M2 phenotypes within the tumor microenvironment. Previous studies have shown that high levels of M1 macrophages predict better prognosis while increasing levels of M2 macrophages indicate poor outcomes (Ma et al., 2010; Yuan et al., 2014; Mei et al., 2016). Liu et al. (2020b) summarized that the high infiltrating levels of M2 macrophages and total tumor-associated macrophages might be negative prognostic factors for patients with GC. Second, we found that the high abundance levels of T cells follicular helper were associated with better prognosis in the low-risk group, and we speculate that it may be due to the antitumor effect of T cells follicular helper. Niogret et al. (2021) provided evidence that T cells follicular helper produce interleukin-21, which exerts an antitumor immune effect through CD8^+^/Tfh crosstalk. A recent study has revealed the important role of T cells follicular helper in mediating antitumor cellular immunity *via* secreting CXCL13 and IL-21 in pancreatic ductal adenocarcinoma (Lin et al., 2021). Third, high-density neutrophils were demonstrated to correlate with poor prognosis of GC patients in the high-risk group. Consistent with our results, Shan et al. (2021) reported that neutrophils were significantly higher in tumor tissues than in normal tissues and were positively associated with tumor progression but negatively correlated with GC patient survival. Above all, the correlation between immune cells infiltration and the identified signature would reflect the differences of immune cells infiltration with different clinical prognosis, and our signature might be a superior prediction for immune infiltration and therapy intervention.

The TIDE algorithm was employed to estimate the underlying treatment efficacy of immunotherapy through the subclass mapping method, which integrated and modeled data from 189 human cancer studies containing a total of 33,197 samples (Jiang et al., 2018). Jiang et al. reported and validated that the TIDE prediction score to access the tumor immune evasion could serve as a stable and reliable surrogate biomarker to predict ICB response, and the utilization of the TIDE score on the prediction of immunotherapeutic efficiency has also been extensively documented (Wang et al., 2021a; Wang et al., 2021b; Zhou et al., 2021). The 18-gene tumor inflammation signature (TIS) enriches the response to PD-1 checkpoint blockade and is also measured for correlation with suppressed adaptive immune response and survival (Dou et al., 2021; Schlam et al., 2021). Herein, both the TIDE and TIS scores were generated. To further validate our signature, we utilized the TIDE and TIS scores in the high- and low-risk groups. Time-dependent ROC curves and AUCs were plotted to determine the clinical utility of the signature by comparing the TIDE and TIS scores. The AUC value for the eight-gene–based signature was 0.710. The results suggested that the immunotherapeutic value of our signature was better than that of the TIDE score (0.518) and TIS score (0.452). In addition, the AUCs of the risk score for predicting 1-, 2-, and 3-year OS were 0.675, 0.682, and 0.710, respectively. The abovementioned findings indicate that our signature has great predictive power.

Nonetheless, there are several major weaknesses in the present study that should be further improved. First, our signature was validated as a prognostic model using the GSE84437 data set as a testing cohort, which showed satisfying efficacy, but multiple large external data sets and effective experimental methods are still needed to further validate the biological role of this risk score model IRG signature in GC. Next, we did not explore the function and mechanisms of the eight DEIRGs underlying the signature, which was significantly correlated with survival, and the underlying mechanism of the eight genes in GC needs to be clarified in depth. Finally, the guidelines for the clinical practice of our IRG signature need to be further defined.

To sum up, our study constructed a novel prognostic signature composed of eight DEIRGs based on the tumor immune microenvironment, which could improve the prediction of the prognosis and immunotherapeutic efficiency for GC patients and might provide novel therapeutic targets for precision oncology in clinical practice.

## Data Availability

The data sets presented in this study can be found in online repositories. The names of the repository/repositories and accession number(s) can be found in the article/Supplementary Material.
