# Supplementary material for: Development of a Novel Immune-Related Gene Signature to Predict Prognosis and Immunotherapeutic Efficiency in Gastric Cancer
Source: Front Genet. 2022 May 27;13:885553. doi: 10.3389/fgene.2022.885553 (PMC9186121; doi:10.3389/fgene.2022.885553)
Supplement: Supplementary file 1 [file Table1.DOCX]

Supplementary Table 1. The TMB in our GC samples

| id | TMB |
| --- | --- |
| TCGA-FP-A4BF | 1.868421 |
| TCGA-D7-A74B | 0.078947 |
| TCGA-HU-A4GH | 11.15789 |
| TCGA-VQ-A8E7 | 1.947368 |
| TCGA-D7-A4YT | 5.552632 |
| TCGA-FP-8210 | 0.026316 |
| TCGA-BR-8365 | 0.368421 |
| TCGA-VQ-A8PZ | 1.368421 |
| TCGA-IN-A7NR | 1 |
| TCGA-CD-5802 | 0.947368 |
| TCGA-IN-AB1V | 0.868421 |
| TCGA-BR-8686 | 1.263158 |
| TCGA-RD-A7C1 | 5.157895 |
| TCGA-BR-8373 | 1.552632 |
| TCGA-VQ-A94O | 1.026316 |
| TCGA-VQ-AA6K | 1.421053 |
| TCGA-VQ-A91W | 0.868421 |
| TCGA-BR-6455 | 1.710526 |
| TCGA-BR-6803 | 0.210526 |
| TCGA-HU-A4HB | 0.052632 |
| TCGA-VQ-A91E | 15.18421 |
| TCGA-BR-8295 | 1.131579 |
| TCGA-VQ-A8E3 | 15.76316 |
| TCGA-BR-8080 | 1.789474 |
| TCGA-HU-A4H2 | 3.394737 |
| TCGA-BR-A453 | 0.184211 |
| TCGA-BR-8284 | 3.973684 |
| TCGA-CD-8530 | 1.157895 |
| TCGA-CG-4476 | 1.947368 |
| TCGA-VQ-A91Y | 0.921053 |
| TCGA-IN-A6RJ | 1.684211 |
| TCGA-CD-5804 | 1.447368 |
| TCGA-IN-A7NU | 1.684211 |
| TCGA-IN-A6RO | 5.657895 |
| TCGA-KB-A93J | 3.342105 |
| TCGA-VQ-A8DZ | 1.447368 |
| TCGA-D7-8575 | 1.184211 |
| TCGA-R5-A7ZF | 0.868421 |
| TCGA-F1-A72C | 0.842105 |
| TCGA-BR-8077 | 1.973684 |
| TCGA-VQ-A8P5 | 4.105263 |
| TCGA-D7-A74A | 3.052632 |
| TCGA-BR-8380 | 0.842105 |
| TCGA-BR-A4CS | 2.894737 |
| TCGA-D7-6527 | 4.026316 |
| TCGA-HU-A4H4 | 9.473684 |
| TCGA-BR-7716 | 2.289474 |
| TCGA-CD-5801 | 5.315789 |
| TCGA-HU-A4GQ | 41.57895 |
| TCGA-HU-A4GU | 30.42105 |
| TCGA-HU-A4HD | 3.526316 |
| TCGA-CG-5730 | 2.842105 |
| TCGA-HU-8244 | 2.394737 |
| TCGA-CG-4440 | 2.684211 |
| TCGA-CG-4462 | 0.657895 |
| TCGA-VQ-A91X | 4.342105 |
| TCGA-HF-7136 | 1.763158 |
| TCGA-VQ-A8PF | 1.078947 |
| TCGA-CD-A486 | 2.026316 |
| TCGA-BR-8363 | 26.60526 |
| TCGA-BR-8297 | 1.605263 |
| TCGA-HF-7134 | 6.078947 |
| TCGA-BR-4357 | 4.289474 |
| TCGA-CG-5719 | 0.947368 |
| TCGA-HU-A4H8 | 26.57895 |
| TCGA-KB-A93G | 0.815789 |
| TCGA-BR-A4IZ | 0.026316 |
| TCGA-HU-A4GC | 2.5 |
| TCGA-BR-6453 | 2.815789 |
| TCGA-RD-A7BT | 4.5 |
| TCGA-D7-A6ET | 0.078947 |
| TCGA-VQ-AA6F | 0.605263 |
| TCGA-BR-8589 | 19.97368 |
| TCGA-VQ-A94R | 5.973684 |
| TCGA-CD-A48A | 4.710526 |
| TCGA-HU-A4H6 | 1.289474 |
| TCGA-HF-7131 | 1.842105 |
| TCGA-HU-8238 | 3.631579 |
| TCGA-BR-8367 | 1.078947 |
| TCGA-D7-A4Z0 | 5.789474 |
| TCGA-BR-4369 | 3.131579 |
| TCGA-D7-A748 | 0.289474 |
| TCGA-CG-4449 | 1.763158 |
| TCGA-BR-8682 | 1.315789 |
| TCGA-BR-6457 | 1.447368 |
| TCGA-CG-4474 | 1.710526 |
| TCGA-FP-A8CX | 1.236842 |
| TCGA-BR-8286 | 2.5 |
| TCGA-D7-8573 | 1.552632 |
| TCGA-3M-AB46 | 3.605263 |
| TCGA-VQ-A91Q | 1.973684 |
| TCGA-VQ-A91U | 2.789474 |
| TCGA-B7-A5TI | 13.23684 |
| TCGA-IN-A6RR | 2.868421 |
| TCGA-BR-7723 | 3.052632 |
| TCGA-VQ-A94U | 3.236842 |
| TCGA-HU-A4GX | 28.39474 |
| TCGA-HF-7132 | 25.18421 |
| TCGA-VQ-A8P3 | 6.078947 |
| TCGA-EQ-5647 | 1.5 |
| TCGA-MX-A663 | 1.210526 |
| TCGA-D7-6518 | 1.026316 |
| TCGA-CG-4466 | 3.921053 |
| TCGA-BR-8059 | 14.05263 |
| TCGA-D7-5579 | 2.263158 |
| TCGA-HU-A4GY | 0.473684 |
| TCGA-KB-A93H | 3.947368 |
| TCGA-BR-4370 | 23.31579 |
| TCGA-CG-4305 | 26.47368 |
| TCGA-D7-6525 | 3.131579 |
| TCGA-VQ-A8PK | 1.710526 |
| TCGA-HU-8245 | 1.263158 |
| TCGA-IN-8462 | 2.421053 |
| TCGA-BR-A4J9 | 0.473684 |
| TCGA-VQ-A91S | 4.842105 |
| TCGA-CD-8529 | 3.657895 |
| TCGA-BR-8683 | 2.842105 |
| TCGA-HU-A4H5 | 5.789474 |
| TCGA-VQ-A91V | 5.263158 |
| TCGA-BR-A4PE | 4.394737 |
| TCGA-BR-8590 | 1.394737 |
| TCGA-VQ-AA68 | 3.763158 |
| TCGA-BR-4280 | 16.57895 |
| TCGA-RD-A8N9 | 2.184211 |
| TCGA-D7-8574 | 0.078947 |
| TCGA-VQ-A94P | 0.421053 |
| TCGA-HF-A5NB | 31.89474 |
| TCGA-BR-6452 | 70 |
| TCGA-BR-8687 | 3.052632 |
| TCGA-VQ-A8PD | 0.736842 |
| TCGA-BR-8285 | 1.605263 |
| TCGA-FP-7916 | 1.5 |
| TCGA-HU-A4G9 | 17.73684 |
| TCGA-MX-A5UG | 0.184211 |
| TCGA-IN-7806 | 1.473684 |
| TCGA-IN-AB1X | 2.421053 |
| TCGA-CD-5798 | 0.921053 |
| TCGA-IN-A6RS | 2.105263 |
| TCGA-BR-7717 | 2.552632 |
| TCGA-VQ-AA6I | 2.052632 |
| TCGA-IN-7808 | 1.894737 |
| TCGA-BR-8361 | 35.34211 |
| TCGA-CD-A487 | 3.5 |
| TCGA-BR-7959 | 1.815789 |
| TCGA-CG-4465 | 13.5 |
| TCGA-FP-7998 | 0.868421 |
| TCGA-D7-6528 | 5.710526 |
| TCGA-BR-6454 | 2.526316 |
| TCGA-IN-A6RP | 3.026316 |
| TCGA-BR-4201 | 32.34211 |
| TCGA-CG-4306 | 24.73684 |
| TCGA-D7-8572 | 3.394737 |
| TCGA-BR-8078 | 36.13158 |
| TCGA-CG-4477 | 2.894737 |
| TCGA-VQ-A8PB | 17.89474 |
| TCGA-D7-A6EZ | 5.815789 |
| TCGA-BR-8364 | 0.210526 |
| TCGA-VQ-A8P2 | 144.7895 |
| TCGA-HF-7133 | 1.605263 |
| TCGA-BR-8291 | 0.473684 |
| TCGA-D7-6526 | 3.868421 |
| TCGA-BR-7722 | 0.763158 |
| TCGA-MX-A5UJ | 30.26316 |
| TCGA-ZQ-A9CR | 1.736842 |
| TCGA-VQ-AA6B | 1.578947 |
| TCGA-B7-A5TK | 0.842105 |
| TCGA-HU-A4GP | 2.894737 |
| TCGA-VQ-A8E2 | 2.052632 |
| TCGA-D7-6524 | 1.526316 |
| TCGA-HU-8243 | 2.447368 |
| TCGA-BR-8679 | 2.842105 |
| TCGA-BR-6802 | 7.763158 |
| TCGA-BR-6458 | 5.447368 |
| TCGA-BR-8366 | 3.026316 |
| TCGA-BR-A4QL | 41.73684 |
| TCGA-D7-A6F0 | 2.315789 |
| TCGA-HU-A4H3 | 20.52632 |
| TCGA-D7-A747 | 0.973684 |
| TCGA-R5-A7ZI | 17.68421 |
| TCGA-CG-4304 | 1.394737 |
| TCGA-BR-A452 | 2.157895 |
| TCGA-BR-A4CQ | 3.052632 |
| TCGA-BR-8368 | 22.05263 |
| TCGA-BR-6456 | 0.815789 |
| TCGA-B7-A5TN | 1.605263 |
| TCGA-BR-A4QI | 2.5 |
| TCGA-D7-6521 | 1.184211 |
| TCGA-BR-8296 | 0.894737 |
| TCGA-VQ-A8DT | 2.789474 |
| TCGA-BR-8372 | 27.68421 |
| TCGA-D7-6820 | 1.421053 |
| TCGA-CG-4301 | 0.973684 |
| TCGA-CD-5803 | 0.526316 |
| TCGA-VQ-A8P8 | 1.026316 |
| TCGA-VQ-AA64 | 5.578947 |
| TCGA-CG-4475 | 1.157895 |
| TCGA-BR-8371 | 0.078947 |
| TCGA-D7-8576 | 0.815789 |
| TCGA-BR-4267 | 1.815789 |
| TCGA-D7-6522 | 0.026316 |
| TCGA-MX-A666 | 1.631579 |
| TCGA-VQ-AA6D | 13.05263 |
| TCGA-VQ-AA6A | 1.815789 |
| TCGA-BR-A4CR | 1.236842 |
| TCGA-BR-7901 | 1.710526 |
| TCGA-VQ-A8DL | 3.473684 |
| TCGA-D7-8578 | 1.052632 |
| TCGA-CD-A4MI | 18.78947 |
| TCGA-BR-7715 | 2.473684 |
| TCGA-VQ-A8PT | 31.44737 |
| TCGA-CG-5720 | 1.394737 |
| TCGA-3M-AB47 | 2.289474 |
| TCGA-BR-6801 | 0.815789 |
| TCGA-BR-6709 | 0.605263 |
| TCGA-HU-A4G6 | 1.736842 |
| TCGA-EQ-8122 | 2.868421 |
| TCGA-BR-7958 | 3.289474 |
| TCGA-B7-A5TJ | 3.894737 |
| TCGA-BR-8487 | 64.18421 |
| TCGA-BR-A4PF | 2.368421 |
| TCGA-HU-A4G8 | 34.15789 |
| TCGA-VQ-A923 | 1.710526 |
| TCGA-BR-8592 | 0.315789 |
| TCGA-VQ-A8DV | 1.5 |
| TCGA-BR-4184 | 69.89474 |
| TCGA-RD-A7BW | 0.447368 |
| TCGA-BR-8360 | 19.73684 |
| TCGA-CD-5799 | 0.631579 |
| TCGA-D7-6815 | 2.342105 |
| TCGA-IN-A7NT | 2.421053 |
| TCGA-R5-A7O7 | 2.078947 |
| TCGA-BR-6563 | 0.605263 |
| TCGA-BR-8483 | 2.473684 |
| TCGA-HU-8608 | 1.815789 |
| TCGA-BR-A44U | 1.684211 |
| TCGA-VQ-A927 | 0.157895 |
| TCGA-R5-A805 | 2.552632 |
| TCGA-HU-A4GT | 29.34211 |
| TCGA-KB-A6F7 | 1.631579 |
| TCGA-VQ-A91A | 2.473684 |
| TCGA-BR-7851 | 30.31579 |
| TCGA-BR-A4J6 | 0.684211 |
| TCGA-VQ-A91D | 52.21053 |
| TCGA-D7-A6EY | 26.47368 |
| TCGA-CD-8526 | 1.052632 |
| TCGA-CD-8535 | 5.421053 |
| TCGA-VQ-A8PP | 31.84211 |
| TCGA-D7-A4YU | 4.631579 |
| TCGA-D7-8570 | 1.921053 |
| TCGA-CD-8528 | 2.052632 |
| TCGA-BR-A4J8 | 1.236842 |
| TCGA-F1-6875 | 1.868421 |
| TCGA-CG-5728 | 27.60526 |
| TCGA-BR-8591 | 32.81579 |
| TCGA-BR-8060 | 4.052632 |
| TCGA-RD-A8N6 | 3.052632 |
| TCGA-D7-A6EX | 2.210526 |
| TCGA-CG-5726 | 26.65789 |
| TCGA-VQ-AA69 | 1.394737 |
| TCGA-VQ-A8PS | 0.815789 |
| TCGA-BR-8370 | 2.157895 |
| TCGA-CG-5724 | 2.184211 |
| TCGA-D7-6822 | 5.5 |
| TCGA-D7-5577 | 2.763158 |
| TCGA-VQ-A94T | 1.5 |
| TCGA-IN-A6RI | 1.710526 |
| TCGA-D7-8579 | 1.894737 |
| TCGA-BR-6852 | 21.15789 |
| TCGA-BR-A4J1 | 1.289474 |
| TCGA-VQ-A925 | 2.342105 |
| TCGA-HU-8602 | 35 |
| TCGA-HU-A4G2 | 0.842105 |
| TCGA-BR-4256 | 21.34211 |
| TCGA-HJ-7597 | 15.86842 |
| TCGA-BR-8485 | 5.947368 |
| TCGA-BR-6565 | 1.973684 |
| TCGA-CG-5721 | 86.97368 |
| TCGA-IN-A6RL | 6.5 |
| TCGA-RD-A8N1 | 1.552632 |
| TCGA-CD-8524 | 2.973684 |
| TCGA-BR-A4J4 | 1.710526 |
| TCGA-VQ-A8E0 | 2.131579 |
| TCGA-BR-6706 | 2.631579 |
| TCGA-D7-6817 | 1.315789 |
| TCGA-D7-A6F2 | 1.236842 |
| TCGA-F1-6177 | 29.68421 |
| TCGA-HU-8604 | 3.605263 |
| TCGA-RD-A8NB | 9.842105 |
| TCGA-BR-8676 | 1.710526 |
| TCGA-BR-8369 | 2.815789 |
| TCGA-BR-A4IV | 0.078947 |
| TCGA-D7-A4YY | 10.07895 |
| TCGA-CD-8533 | 2.236842 |
| TCGA-CG-5722 | 1.105263 |
| TCGA-BR-7197 | 3.657895 |
| TCGA-RD-A8MW | 1.078947 |
| TCGA-BR-8678 | 2.342105 |
| TCGA-CG-4443 | 1.763158 |
| TCGA-D7-6520 | 1.157895 |
| TCGA-BR-4363 | 13.34211 |
| TCGA-BR-7704 | 7.289474 |
| TCGA-HU-A4GD | 1.526316 |
| TCGA-CG-5717 | 0.342105 |
| TCGA-CG-5733 | 24.28947 |
| TCGA-BR-A4J2 | 0.289474 |
| TCGA-D7-A4YX | 1.710526 |
| TCGA-BR-8690 | 2.473684 |
| TCGA-CG-4437 | 9.447368 |
| TCGA-CD-A48C | 3.815789 |
| TCGA-BR-4188 | 1.842105 |
| TCGA-CG-4300 | 1.578947 |
| TCGA-RD-A7BS | 0.736842 |
| TCGA-HU-A4GN | 20.94737 |
| TCGA-CD-8531 | 4.947368 |
| TCGA-HU-A4G3 | 1.815789 |
| TCGA-VQ-A8PX | 11.36842 |
| TCGA-HU-A4H0 | 3.052632 |
| TCGA-VQ-A928 | 1.263158 |
| TCGA-BR-8381 | 1.447368 |
| TCGA-CG-4444 | 2.921053 |
| TCGA-BR-7703 | 14.05263 |
| TCGA-F1-6874 | 19.97368 |
| TCGA-CG-4455 | 0.684211 |
| TCGA-CG-5734 | 0.684211 |
| TCGA-SW-A7EB | 2.263158 |
| TCGA-CD-5800 | 2.289474 |
| TCGA-BR-4255 | 0.815789 |
| TCGA-BR-8486 | 1.078947 |
| TCGA-BR-6566 | 16.23684 |
| TCGA-CD-A4MJ | 14.89474 |
| TCGA-D7-A6EV | 2.447368 |
| TCGA-CD-8534 | 1.052632 |
| TCGA-FP-A4BE | 33.60526 |
| TCGA-VQ-A8PY | 1.394737 |
| TCGA-CG-4441 | 2.052632 |
| TCGA-BR-7707 | 21.81579 |
| TCGA-BR-8384 | 0.184211 |
| TCGA-BR-4294 | 0.657895 |
| TCGA-BR-7957 | 0.684211 |
| TCGA-BR-6705 | 0.815789 |
| TCGA-BR-4361 | 47.23684 |
| TCGA-FP-7829 | 5.184211 |
| TCGA-BR-4279 | 0.868421 |
| TCGA-BR-A4IU | 0.342105 |
| TCGA-CD-A489 | 1.552632 |
| TCGA-EQ-A4SO | 3.947368 |
| TCGA-CG-5732 | 0.605263 |
| TCGA-FP-8211 | 2.210526 |
| TCGA-R5-A804 | 1.105263 |
| TCGA-CG-4460 | 43.05263 |
| TCGA-CG-4442 | 32.05263 |
| TCGA-BR-A4PD | 3 |
| TCGA-BR-4362 | 32.44737 |
| TCGA-F1-A448 | 6.684211 |
| TCGA-VQ-A8PE | 2.342105 |
| TCGA-CG-5727 | 2.684211 |
| TCGA-VQ-AA6G | 2.631579 |
| TCGA-VQ-A8PC | 2.131579 |
| TCGA-BR-4191 | 4.315789 |
| TCGA-BR-A4J7 | 0.315789 |
| TCGA-CD-8527 | 4.736842 |
| TCGA-BR-8382 | 18.92105 |
| TCGA-BR-4292 | 31.92105 |
| TCGA-BR-A4QM | 1.868421 |
| TCGA-CD-8532 | 0.605263 |
| TCGA-CG-5723 | 38.86842 |
| TCGA-VQ-A8PM | 0.815789 |
| TCGA-VQ-A8PJ | 3 |
| TCGA-BR-8081 | 18.23684 |
| TCGA-VQ-A91Z | 2.973684 |
| TCGA-SW-A7EA | 13.44737 |
| TCGA-R5-A7ZE | 2.157895 |
| TCGA-VQ-A8PO | 17.34211 |
| TCGA-VQ-A91K | 29.73684 |
| TCGA-ZA-A8F6 | 1.078947 |
| TCGA-BR-8289 | 4.289474 |
| TCGA-BR-8680 | 133.6579 |
| TCGA-D7-6818 | 3.289474 |
| TCGA-CD-A4MH | 1.473684 |
| TCGA-BR-4257 | 24.15789 |
| TCGA-IP-7968 | 2.263158 |
| TCGA-D7-6519 | 1.105263 |
| TCGA-VQ-AA6J | 2.157895 |
| TCGA-IN-8663 | 3.421053 |
| TCGA-BR-6707 | 1.315789 |
| TCGA-CG-4469 | 6.789474 |
| TCGA-HU-A4GF | 2.684211 |
| TCGA-VQ-A91N | 2.052632 |
| TCGA-BR-4371 | 3.157895 |
| TCGA-BR-4253 | 1.789474 |
| TCGA-FP-A9TM | 2.473684 |
| TCGA-FP-8631 | 1.947368 |
| TCGA-D7-A4YV | 19.39474 |
| TCGA-BR-A4IY | 2.605263 |
| TCGA-BR-8058 | 0.657895 |
| TCGA-FP-8099 | 2.342105 |
| TCGA-D7-5578 | 4.210526 |
| TCGA-VQ-A922 | 2.105263 |
| TCGA-VQ-A8PU | 2.342105 |
| TCGA-BR-4187 | 0.368421 |
| TCGA-HU-8249 | 3.105263 |
| TCGA-CG-5716 | 0.078947 |
| TCGA-CD-5813 | 2.921053 |
| TCGA-BR-A4J5 | 0.973684 |
| TCGA-BR-8588 | 1.868421 |
| TCGA-CG-4438 | 2.815789 |
| TCGA-B7-5816 | 17.92105 |
| TCGA-CD-8525 | 1.526316 |
| TCGA-R5-A7ZR | 1.473684 |
| TCGA-CG-5718 | 1.684211 |
| TCGA-HU-8610 | 1 |
| TCGA-VQ-A8PH | 4.026316 |
| TCGA-CD-8536 | 20.18421 |
| TCGA-BR-6564 | 0.368421 |
| TCGA-BR-8484 | 0.921053 |
| TCGA-CG-4436 | 2.210526 |
| TCGA-B7-5818 | 8.026316 |
| TCGA-CD-A4MG | 49.47368 |
| TCGA-BR-8677 | 0.789474 |
| TCGA-VQ-A924 | 30.65789 |
| TCGA-RD-A8MV | 1.342105 |
| TCGA-BR-4183 | 0.842105 |
| TCGA-BR-4366 | 2.526316 |
| TCGA-IN-A6RN | 1.815789 |
| TCGA-RD-A8N5 | 0.368421 |
| TCGA-VQ-A92D | 2.421053 |
| TCGA-BR-7196 | 0.894737 |
| TCGA-CG-5725 | 2.289474 |
| TCGA-FP-7735 | 0.736842 |
| TCGA-BR-4368 | 27.15789 |
| TCGA-VQ-A8DU | 3.157895 |
