# Supplementary figures and images for: Development of a Novel Immune-Related Gene Signature to Predict Prognosis and Immunotherapeutic Efficiency in Gastric Cancer
Source: Front Genet. 2022 May 27;13:885553. doi: 10.3389/fgene.2022.885553 (PMC9186121; doi:10.3389/fgene.2022.885553)

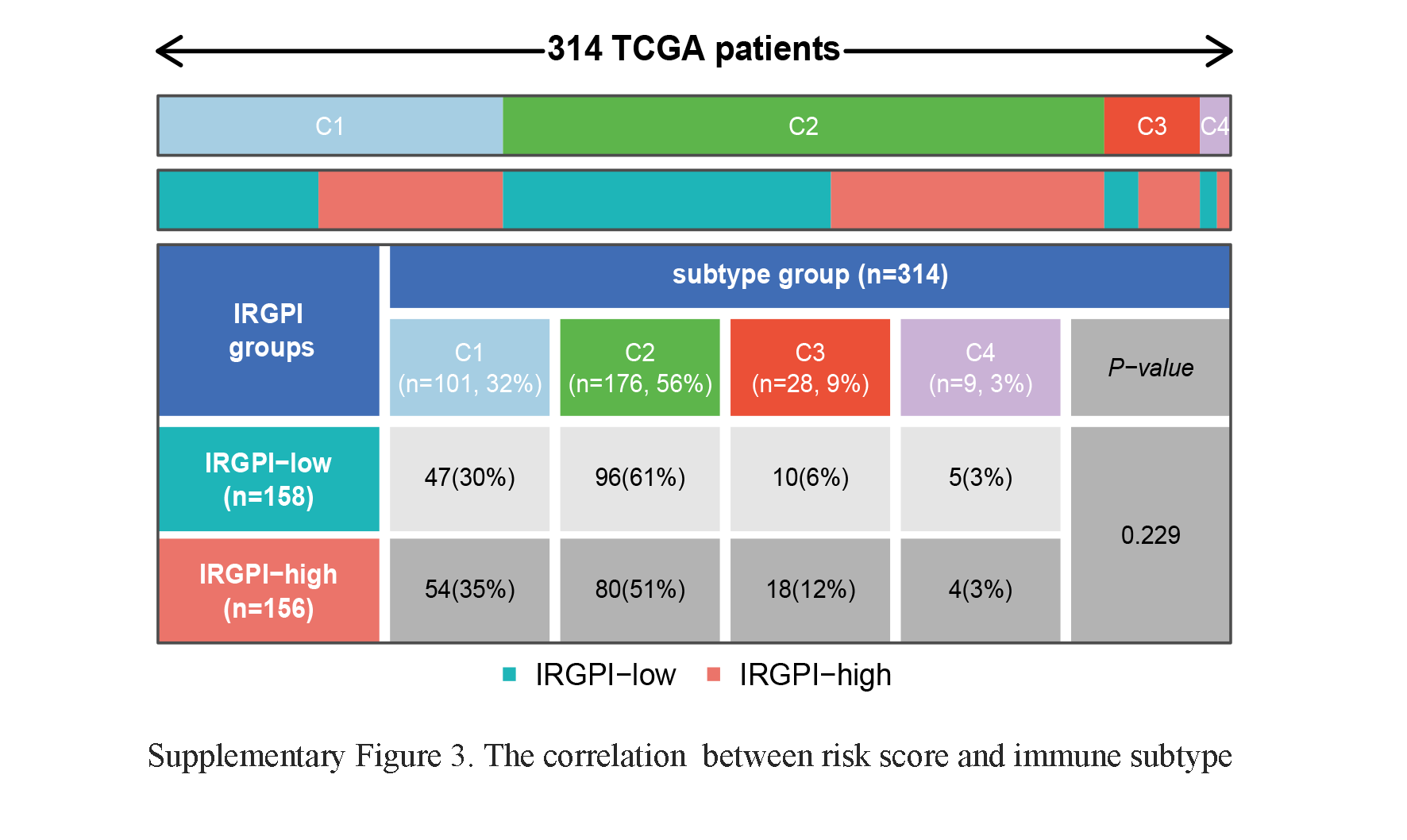

Supplement: Supplementary file 2 [file Image3.TIF]

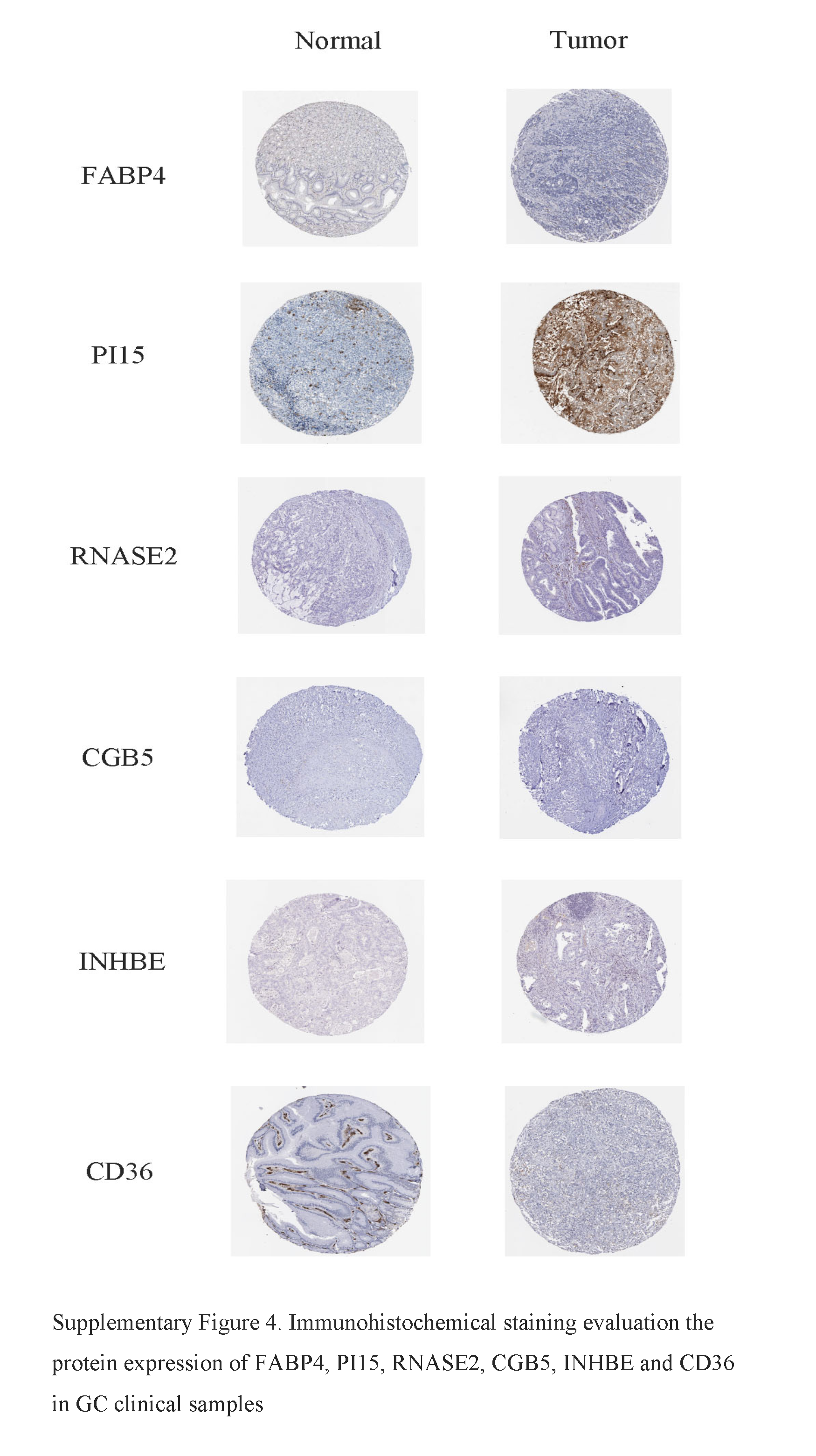

Supplement: Supplementary file 3 [file Image4.TIF]

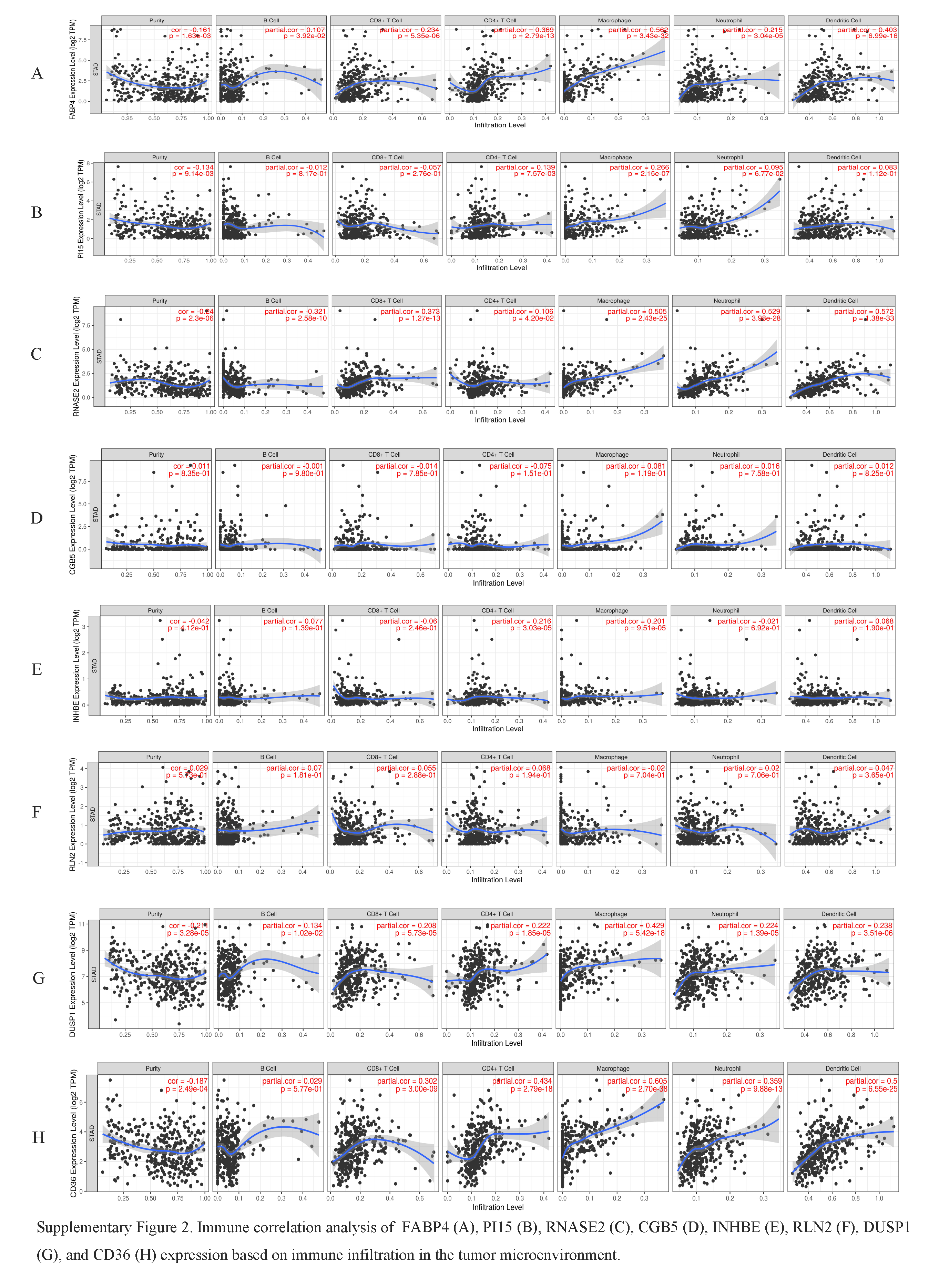

Supplement: Supplementary file 4 [file Image2.TIF]

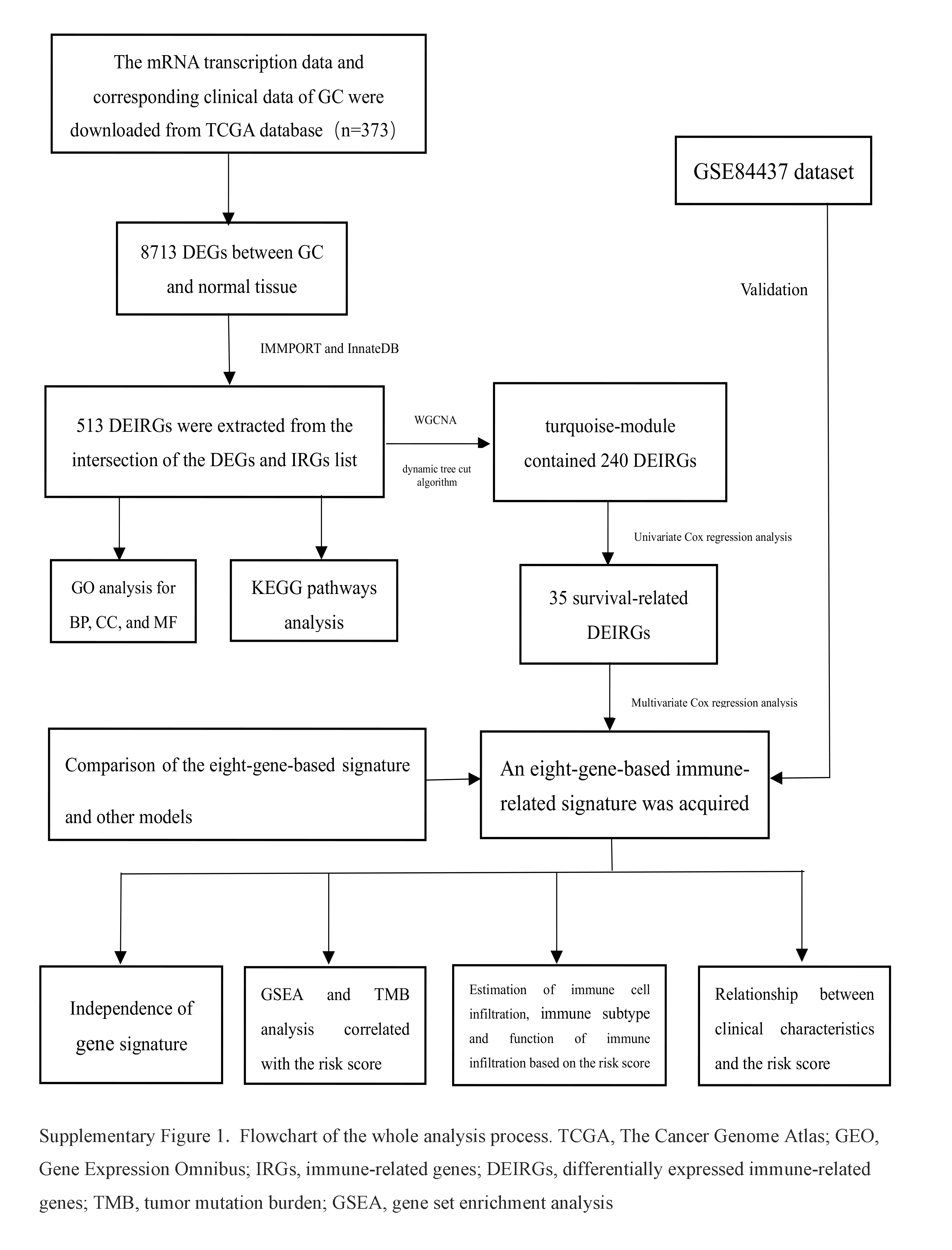

Supplement: Supplementary file 5 [file Image1.TIF]

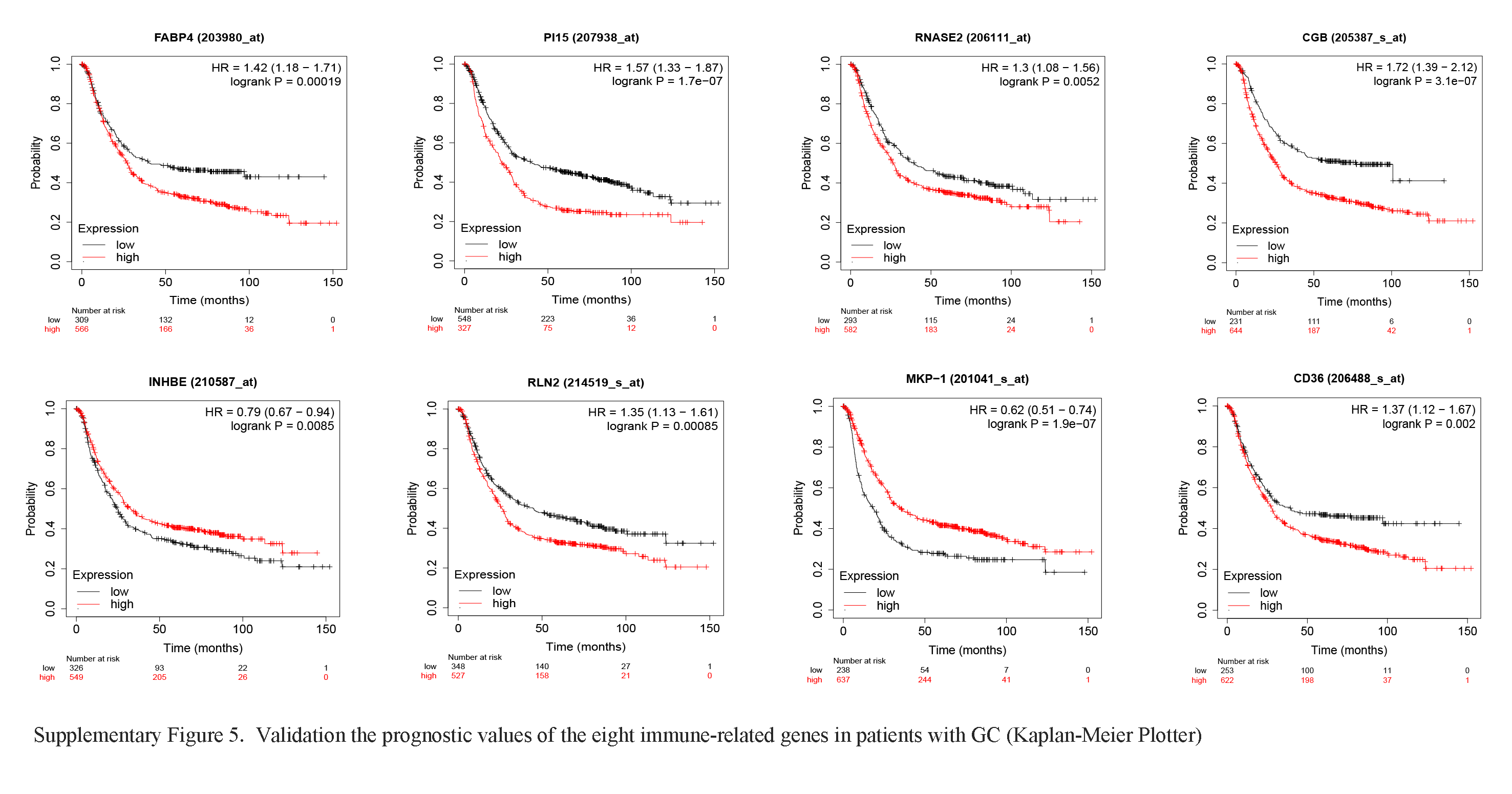

Supplement: Supplementary file 7 [file Image5.TIF]
