# Supplementary material for: Development of a Novel Immune-Related Gene Signature to Predict Prognosis and Immunotherapeutic Efficiency in Gastric Cancer
Source: Front Genet. 2022 May 27;13:885553. doi: 10.3389/fgene.2022.885553 (PMC9186121; doi:10.3389/fgene.2022.885553)
Supplement: Supplementary file 6 [file Table2.DOCX]

**Working Flow**

1. TCGA transcriptional data download
2. Transcriptional data processing
3. ID conversion
4. TCGA clinical data download
5. Clinical data sorting
6. Acquisition of immune related genes
7. GEO Data download
8. GEO Data Annotation
9. TCGA Mutation data download
10. Calculate tumor mutation burden
11. Difference analysis
12. GO Enrichment analysis
13. KEGG Enrichment analysis
14. WGCNA
15. Gene-gene interaction network Analysis
16. Identification of Co-expression Modules
17. Merge expression data and survival data TCGA
18. Merge expression data and survival data GEO
19. Identification of prognosis related genes
20. tumor mutation burden of prognosis related genes
21. Establishment of the immune-related risk signature
22. Survival analysis
23. Univariate and multivariate Cox analyses
24. GSEA
25. File preparation of waterfall plot
26. Waterfall plot
27. Immune cell infiltration
28. Differential analysis of immune cells
29. Survival analysis of immune cells
30. Difference analysis of immune related functions
31. Survival analysis of immune related functions
32. Tumor mutation burden analysis
33. Analyses of correlation between risk score and clinical characteristics
34. The correlation between risk score and immune subtype
35. Immune escape and immunotherapy
36. Model comparison
